# Lagged-price reimbursement contracts: The impact of medicare Part B on pharmaceutical price growth

**DOI:** 10.1016/j.jpubeco.2026.105595

**Published:** 2026-02-20

**Authors:** Angelique Acquatella, Keith Marzilli Ericson, Amanda Starc

**Affiliations:** a Toulouse School of Economics, Toulouse, France; b Questrom School of Business, Boston University, and NBER, MA, Boston, United States of America; c Kellogg School of Management, Northwestern University, Evanston, Cook

**Keywords:** Dynamic pricing, Government contracting, Pharmaceuticals, Payment policy, I11, I13, I18, H32, H57

## Abstract

We examine lagged-price cost-plus reimbursement contracts, focusing on Medicare Part B’s payment for physician-administered drugs. While previous research has shown that Part B increased launch prices, we estimate its effect on later prices and find that lagged-price reimbursement *lowers* prices in later periods. Drugs more exposed to Medicare reimbursement have lower price growth (net of rebates): a drug with above median Part B exposure has a 10% lower price after 3 years than a below median exposure drug that launched at the same price. The effect is larger for newly approved molecules, which face less competition.

## 1. Introduction

Governments pay for goods and services from private firms, ranging from defense contractors to healthcare providers. When contracting with the government, prices are rarely determined by a market mechanism, which can lead to distortions. In an efficient market, prices signal firms’ production costs and consumers’ willingness to pay. Absent such an information aggregating mechanism, governments frequently use cost-plus contracts. Absent good information on costs, the government may use proxies, including past prices. The combination of cost-plus contracting and dynamic incentives can amplify or dampen distortions, especially when firms price strategically: the input price set by the firm today will affect the future reimbursement that contractors buying these inputs will receive from the payer.

This paper estimates the effects of lagged-price cost-plus reimbursement on dynamic incentives for price setting. We focus on physician-administered drugs covered by Medicare Part B, which includes anti-cancer/chemotherapy drugs and immunosuppressives. However, lagged-price cost-plus procurement contracts also appear in other markets. For instance, in construction contracting, a producer sets a price for a construction-related input (e.g., asphalt), a construction contractor purchases that input, and– if the contract contains an economic price adjustment clause– the government makes additional payments to contractors if an index of prices is higher than forecasted.^1^ Lagged-price contracts also inadvertently appear in European pharmaceutical contracting that uses external reference pricing contracts, as these condition on observed prices which have already been agreed upon by the time of negotiation (Maini and Pammolli, 2023).

Payment for prescription drugs is particularly controversial, as the government (via Medicare) is a major purchaser from pharmaceutical firms that often hold a monopoly on the drug. In Medicare Part B, which covers physician-administered drugs, the government pays physicians using cost-plus reimbursement based on lagged prices. However, widespread concern about rising drug prices has driven proposals to change how drugs are paid for and recent policy reforms in which the government will directly negotiate drug prices.^2^ Moreover, in addition to affecting drug spending, Medicare Part B payment policy could have important consequences for enrollee health. Medicare is a major payer for cancer care in the US, and Part B drugs are a major source of revenue for oncology practices.

Part B has a buy-and-bill policy, in which hospitals and clinics purchase drugs (either on their own, or as part of a group purchasing organization). Medicare pays physicians when they deliver these drugs based on lagged average cost (from two quarters ago) plus a percentage markup. The policy has different incentives than either a simple fixed price or cost-plus contract (see Bajari and Tadelis (2001) on these contract forms). In particular, since physician margin is increasing in lagged price, higher prices may ultimately lead physicians to prescribe *more*, as they result in higher reimbursement. While the introduction of the current Part B payment policy has been linked to higher drug prices at launch compared to previous policy (Howard et al., 2015; Ridley and Lee, 2020), it is unknown how Part B affects changes in prices over time.

We first develop a conceptual framework to understand the economic forces that could generate the observed pricing patterns, which contrast with “invest-then-harvest” pricing that is commonly observed in markets (e.g., Farrell and Shapiro (1988); Ericson (2014)). We model the key features of Medicare payment for physician-administered drugs: pharmaceutical firms set prices, physicians buy the drug on behalf of patients and choose how much to consume, and Medicare reimburses physicians based on lagged market average prices. In our theoretical model, pharmaceutical firms account for changes in future reimbursement when setting prices. Physician demand is affected by both current price and reimbursement, the difference between which is their margin. But because reimbursement levels affect not only physician reimbursement but also patients’ level of cost-sharing, the model allows price and reimbursement to have different impacts on demand. We show that lagged-price cost-plus reimbursement, as implemented in Part B, can lead to both higher initial prices *and* lower prices relative to launch in later periods.

We then examine empirically how Part B’s payment policy affects price changes over time during the period 2006–2019. Our identifying variation comes from drugs that are more or less exposed to Part B: the share of expenditures for a drug that comes via Medicare Part B, as opposed to private insurers. A similar research design is used by Yurukoglu et al. (2017) to show that exposure to Part B led to shortages in the generic market.^3^ We observe average prices net of rebates. Our identification strategy includes a drug fixed effect, so it does not rely on Medicare market share not being correlated with drug value or demand.

For a drug whose Medicare market share at launch is above the median, we estimate that prices 3 years after launch are at least 10% lower than those of a drug with below median exposure that launched at the same price. The effect is larger for newly approved molecules, which face less competition. Moreover, to the extent that private insurers partially follow Medicare’s pricing, our results will underestimate Medicare’s effect.

Previous literature showed that physician-administered drug prices at launch have been increasing over time (Howard et al. (2015) on anti-cancer drugs), and changes to Part B reimbursement in 2006 led to higher launch prices (Ridley and Lee, 2020). We show that, following launch, more exposure to Part B payment led to slower price growth. We further document that the dynamic impact dampens but likely does not erase the overall upward pressure on prices generated by the Medicare program.

Our paper is related to a large literature that explores the impact of contracting and procurement rules in healthcare (e.g., Gaynor et al. (2023); Decarolis (2015)), construction (e.g., Bosio et al. (2022); Krasnokutskaya and Seim (2011)), and telecommunications (e.g., Kang and Miller (2022)). However, many of these papers do not examine the effects on how prices evolve over time (for an exception, see Ji and Rogers (2023)). Our paper examines these dynamic forces theoretically and empirically.

## 2. Institutional setting

The Medicare program provides health insurance to elderly and disabled individuals in the United States. Part B covers outpatient care, including drugs administered by physicians. The majority of Part B drug payments are for services rendered in physician office settings, which are very different from the outpatient drugs covered by Part D. Spending on Medicare Part B drugs totaled $37.1 billion in 2019, which is about one-fifth the size of spending on Part D drugs (Medicare Payment Advisory Commission, 2022). The top ten drugs ranked by Medicare Part B expenditures constitute about 40% of Part B drug spending.

Since 2005, Medicare has reimbursed providers based on average sales price (ASP) (Jacobson et al., 2010; Yurukoglu et al., 2017; Ridley and Lee, 2020). The provider pays a price to the manufacturer or distributor. Medicare constructs the ASP as a manufacturer-reported, volume-weighted average of U.S. sales prices for a drug, net of most discounts and rebates. The provider is then reimbursed at lagged ASP times a multiplier, and payments received typically correspond to 106% of the ASP from two quarters ago.^4^ The out-of-pocket costs for the patient are 20% of the reimbursed amount in the form of coinsurance; the Medicare program covers the remaining 80%.^5^ Initial period reimbursement cannot rely on lagged prices, and is set at a markup over the “list” price, either Wholesale Average Cost (WAC) plus 6% or Average Wholesale Price (AWP) minus 5%. WAC is a list price reported by hospitals for drugs acquired through drug wholesalers. AWP is also a list price, reported by drug wholesalers. As noted by Ridley and Lee (2020), list prices at launch may be artificially high in these markets.

The Part B reimbursement system is controversial for several reasons. First, it is difficult for any administered price system to capture marginal costs. While some worry about overpayment, particularly for biologics (Morton and Boller, 2017), others note that government policy can put a financial strain on providers (Polite et al., 2015). Lagged-price cost-plus reimbursement additionally creates incentives that may affect provider treatment decisions and the lifetime profitability of drugs (Medicare Payment Advisory Commission, 2016a). Paying a percentage add-on over average price might give physicians an incentive to prescribe more expensive drugs, insofar as these yield the physician a higher margin. While physicians also receive drug administration fees under Medicare Part B, for many of these physicians, drug reimbursements for these drugs constitute a substantial share of their revenue. Financial incentives for physicians may be particularly strong in oncology because average drug margins for chemotherapy can range anywhere from a few cents to $2000 for a single dose.

To build intuition for our results, consider how the Part B reimbursement rules may affect a monopolist’s pricing incentives (62% of drugs covered under Part B have a monopoly manufacturer). In the absence of lagged-price reimbursement, a pharmaceutical firm introducing a new drug into the market may want to enter at a low price to encourage physicians to adopt the new treatment. However, with a rising price path, physicians will buy at high prices but get reimbursed based on previous lower prices, giving them lower margin. This is likely to reduce the quantity demanded by physicians from the firm.

Because the pharmaceutical monopolist can control future reimbursement through its choice of current prices, the firm has an incentive to begin with high prices but lower them over time to ensure that Medicare reimburses physicians at a high rate and to give physicians higher margins in future periods. These pricing incentives are exactly the opposite of “invest-then-harvest” pricing, in which firms enter at low prices and raise them in later periods.

## 3. Conceptual framework

We develop a stylized model of monopolist pricing under lagged-price reimbursement to interpret our empirical findings. In the model, some drug purchases are reimbursed by the government with lagged-price reimbursement, and the remainder are reimbursed by private insurers at an independently determined rate. The model shows the conditions under which Medicare’s lagged-price reimbursement will lead to slower price growth or declining prices over time.

### 3.1. Demand

Consider a two period (t=1,2) model of pharmaceutical pricing for a drug.^6^ Pharmaceutical firms set a single national price $p_{t}$ each period.^7^ Physicians purchase drugs directly from the pharmaceutical firm; we abstract away from intermediaries, such as group purchasing organizations. Insurers reimburse physicians for their drug purchases.

The fraction of patients with Medicare (M) insurance is μ, with the remainder in private insurance (P). The Medicare reimbursement rate, $r_{Mt}$, is a function of the average sale price in the previous period times a multiplier (1+A). That is, $r_{Mt}=(1+A)p_{t-1}$ with A>0, for t>1. When t=1 and there is no data available on lagged prices, the Medicare reimbursement is determined outside the model as $r_{M1}$. (In practice, it is based on the manufacturer list price or wholesale price.)

The private reimbursement rate, $r_{Pt}$, is independent of the Medicare rate and is determined outside the model. In order to focus on the pricing dynamics created by lagged-price reimbursement in isolation, we assume private reimbursement remains constant over time, $r_{Pt}=\rho$.

Drug demand depends on physician utility, which consists of both the physician’s margin (the difference between reimbursement and acquisition price) and some weight on patient well-being.^8^ The utility of administering a drug to patient i in time period t is:

$$V_{it}=\underset{\text{physician}\;\text{profits}}{\underbrace{\left(r_{it}-p_{t}\right)}}+\tilde{\lambda }\underset{\text{patient}\;\text{utility}}{\underbrace{\left(h_{it}-oop\left(r_{it}\right)\right)}}$$

where $r_{it}$ is the reimbursement received by the physician, $p_{t}$ is the price the physician pays to acquire the drug, $\tilde{\lambda }$ is the physician’s weight on patient utility, $h_{it}$ is a stochastic health benefit from administering the drug to patient i, and $oop\left(r_{it}\right)$ is the patient out-of-pocket cost, which can depend on reimbursement levels.^9^

To simplify expressions, we define the physician’s *effective* margin for insurer k∈M,P in period t as the difference between weighted reimbursement and the price: $m_{kt}\equiv \lambda r_{kt}-p_{t}$ where the weight on reimbursement $\lambda \equiv \left(1-\tilde{\lambda }\frac{oop\left(r_{kt}\right)}{r_{kt}}\right)$ accounts for both the effect of reimbursement on physician profits and patient cost-sharing. The effective margin is increasing in reimbursement so long as λ>0, which requires the weight placed on physicians’ own reimbursement to outweigh any disutility from higher patient cost-sharing. We assume λ is constant across payers and time and that λ>0.

Quantity demanded is thus a function of the physician’s *effective margin*. The probability that a Medicare patient is prescribed the drug is an increasing function in the Medicare margin: $Q_{M}\left(m_{Mt}\right)\equiv P\left(m_{Mt}+\tilde{\lambda }h_{it}>0\right)$, with $Q_{M}^{\prime }\left(m_{Mt}\right)>0$. A parallel expression defines the probability $Q_{P}$ that a private patient is prescribed the drug. Total quantity prescribed in period t is $Q_{t}\equiv \mu Q_{M}\left(m_{Mt}\right)+(1-\mu )Q_{P}\left(m_{Pt}\right)$, weighting Medicare and private demand by the fraction of Medicare and private patients, respectively.

### 3.2. Price setting

We now consider a pharmaceutical monopolist with constant marginal cost c choosing prices for its drug to maximize profits. Given $r=\left\{r_{M1},r_{M2}\left(p_{1}\right),\rho \right\}$, the pharmaceutical firm chooses a vector of prices $p=\left\{p_{1},p_{2}\right\}$ to maximize

$$\Pi (p;r)=\pi \left(p_{1};r_{M1},\rho \right)+\delta \pi \left(p_{2};r_{M2}\left(p_{1}\right),\rho \right)+\delta^{2}\mu EV_{M}\left(p_{2}\right)+\delta^{2}(1-\mu )EV_{P}$$

where $\pi \left(p_{t};r_{Mt},r_{Pt}\right)\equiv \left(p_{t}-c\right)\left[\mu Q_{M}\left(\lambda r_{Mt}-p_{t}\right)+(1-\mu )Q_{P}\left(\lambda r_{Pt}-p_{t}\right)\right]$ are flow profits in period t. We explicitly allow for continuation profits in future periods. To the extent that firms can affect future Medicare reimbursement via their second period price, Medicare continuation profits are a function of $p_{2}$. Because private reimbursement doesn’t depend on past prices, $EV_{P}$ does not depend on $p_{2}$. For intuition, we will think of the continuation value as near zero (e.g., the firm faces generic entry). We assume that $EV_{M}^{\prime }\left(p_{2}\right)\geq 0$.

Optimal prices will depend on the elasticity of demand with respect to price, as usual. The aggregate semi-elasticity of demand is thus $\eta_{t}\equiv \frac{-dQ_{t}/dp_{t}}{Q_{t}}$. In a single-period static model, the firm would set prices equal to marginal costs plus a markup term based on the inverse semi-elasticity.

Medicare’s lagged-price reimbursement formula links the prices set between periods. The first order conditions for the optimal prices are then:

(1) $$p_{1}=c+\frac{1}{\eta_{1}}+\delta \mu \left(p_{2}-c\right)\underset{\begin{array}{c} \text{effect}\;\text{of}\;↑p_{1}\;\text{on}\;Q_{2}\\ \;\text{demand}\;(\text{through}\;r_{M2}) \end{array}}{\underbrace{(1+A)\lambda \frac{Q_{M}^{\prime }\left(m_{M2}\right)}{-dQ_{1}/dp_{1}}}},$$

(2) $$p_{2}=c+\frac{1}{\eta_{2}}+\underset{\begin{array}{c} \text{effect}\;\text{of}\;↑p_{2}\\ \;\text{on}\;\text{continuation}\;\\ \;\text{profits} \end{array}}{\underbrace{\delta \mu \frac{EV_{M}^{\prime }\left(p_{2}\right)}{-dQ_{2}/dp_{2}}}}.$$

The pricing decisions are not independent across time because $m_{M2}$ depends on $p_{1}$; the optimal launch price depends on the period 2 price and vice versa. The margin in period two will depend on the reimbursement level, which is determined by launch price. In turn, period 1 price depends on the profit margin the firm anticipates in period 2. (Suppose– outside the model– the firm expected to have to price at marginal cost in period 2. Then, there would no longer be an incentive to raise the launch price above the static monopoly price in period 1.)

The first order condition for the choice of launch price shows that the difference between static monopoly pricing and optimal prices depends on the marginal effect of launch prices on future demand. Launch prices affect future demand by changing effective margin to physicians in period 2. This impact on margin depends on the share of Medicare patients μ, the ASP reimbursement multiplier 1+A, and weight λ on reimbursement versus price in physician’s effective margin.

We make a set of technical assumptions formalized in the Theoretical Appendix A. First, we assume that the physician places a positive weight on reimbursement and that the patient out-of-pocket share is constant. Second, we assume that conditions hold such that the pharmaceutical firm’s pricing problem is globally convex and the second-order conditions hold. These conditions require that demand in any given period not be too convex, that the continuation value of future profits is non convex in the period two price (e.g., that the firm cannot make infinite profits in the future by raising current prices), and that the cross-effect of price on future reimbursement is not strong enough to overturn concavity.

Our model focuses on how lagged-price reimbursement shapes the dynamic price path. In practice, other forces also influence price dynamics. For example, general inflation can raise nominal prices over time, independently of reimbursement design. In addition, provider adoption costs can generate interesting dynamic effects. Once providers incur adoption costs, their effective willingness to pay for continued use of the drug increases. As Ridley and Lee (2020) argue, this can induce firms to offer higher provider margins (lower launch prices relative to reimbursement) early on to encourage uptake, with the potential for higher prices in later periods once adoption has occurred. Finally, the dynamic path of prices could be affected by entry of competing therapies, providers learning about drug effectiveness, or manufacturers learning about demand.

### 3.3. Illustrative simulations

**Fig. 1.**
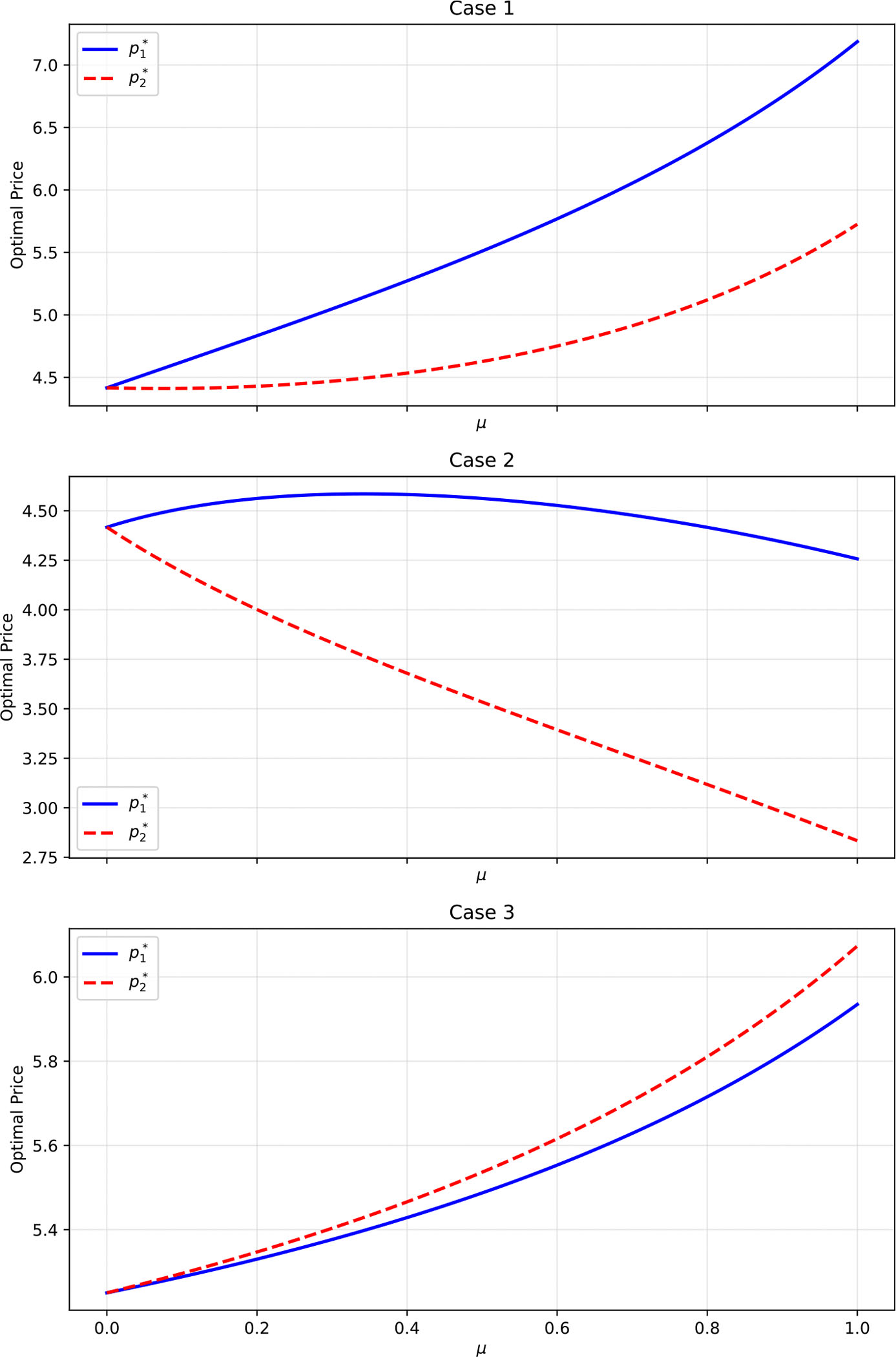
Potential Pricing Dynamics With Lagged-Priced Reimbursement. *Notes*: Authors’ simulations under different parameters. Assumes linear demand with $Q_{k}\left(m_{t}\right)=a_{k}+b_{k}m_{kt}$ for each payer k, no continuation value beyond period 2 and marginal cost =0.5. Case 1 sets $r_{M1}=\rho =5,A=0.06,\delta =1$, and the same demand curve for both Medicare and private, with intercept $a_{M}=a_{P}=0.4$ and slope $b_{M}=b_{P}=0.12$. Case 2 is the same as Case 1 except now A=0.12 and Medicare demand is more price sensitive ($a_{M}=0.08,b_{M}=0.2$). Case 3 sets $r_{M1}=2,\rho =5,A=0.12,\delta =0.7$, and demand such that $a_{M}=a_{P}=0.5$ and slope $b_{M}=b_{P}=0.1$.

To illustrate the ambiguity of lagged-price reimbursement on prices and total lifecycle drug costs, we show a set of simple numerical examples. We do not attempt to calibrate these parameters empirically, given the illustrative two-period model. To see the ambiguity of lagged-price reimbursement on prices and total lifecycle drug costs, we show three separate numerical examples in Fig. 1. Each example has linear demand so for payer $k{\;Q}_{k}\left(m_{t}\right)=a_{k}+b_{k}m_{kt}=a_{k}+b_{k}\left(r_{kt}-p_{t}\right)$. Continuation value after the second period is zero (e.g., due to intense generic competition). In each case, we set private insurer reimbursement ρ to be 10 times marginal cost, which we normalize to be 0.5, and the physician’s weight on reimbursement equal to that on cost (λ=1).

In Fig. 1 Case 1, a higher fraction of patients with Medicare μ raises both first and second period prices. Intuitively, as Medicare’s share grows, it is optimal for firms to set a higher first period price to invest in future Medicare reimbursement; this higher reimbursement then yields a higher second period price.

In Case 2, $p_{2}$ declines in μ, while the effect of μ on $p_{1}$ is non-monotonic. In this case, the add-on is larger (A=0.12) and Medicare demand is more price sensitive than private demand. At low μ, an increase in Medicare’s market share increases the investment motive for future reimbursement, resulting in $p_{1}$ increasing in μ in this region. However, as μ increases, first period demand becomes more price elastic (as Medicare demand is more price sensitive in these simulations), creating a countervailing force lowering $p_{1}$ for higher values of μ, and explaining the non-monotonicity. The result that $p_{2}<p_{1}$ comes from second period demand being more elastic, because Medicare is a larger fraction of second period demand than first period demand as Medicare reimbursement is higher in period 2.

Finally, in Case 3, both periods’ prices increase as μ increases, just as in Case 1. However, $p_{2}>p_{1}$, with this difference becoming larger as μ increases. Here, greater discounting (δ=0.7, relative to δ=1 in Case 1) constrains the firm’s desire to set higher prices in period 1 for future reimbursement, as the firm values those future profits less. However, Medicare reimbursement is high in period 2, so as μ increases, the firm generates substantial revenues from Medicare patients. Its optimal strategy is then to set a higher price for period 2.

While these simulations and our theoretical model focus on a simple two period case, pricing dynamics can be more complex with more periods. We explore simulations with multiple periods in Appendix B. Just as in the main simulations, there is an incentive to set higher average prices early in the product’s life to invest in Medicare reimbursement, followed by lower prices later to harvest profits. However, price changes need not be monotonic over time. For instance, we show a simulation in which the firm optimally and gradually raises prices in a few early periods before later reducing them in later periods; in that example, the firm finds it unprofitable to launch at a very high initial price relative to first-period reimbursement.

### 3.4. Results

Our theoretical results show when the Medicare lagged-price reimbursement system will lead to declining prices over time, as opposed to the flat prices in the private sector. If forces outside the model are leading to price increases over time, this implies that lagged-price reimbursement will lead to slower price growth. Proposition 1 provides an intuitive necessary and sufficient condition for when lagged-price reimbursement will lead to a declining price path: so long as the semi-elasticity of demand in period 2 is not too smaller than in period 1, price will decline over time. The larger the ASP add-on (1+A), the discount rate, and the share of Medicare patients, the more semi-elasticity of demand can differ.

#### Proposition 1.

*The equilibrium price will decrease over time* ($p_{1}>p_{2}$) *if and only if:* $\frac{\eta_{2}}{\eta_{1}}>\left(1+\delta \frac{\mu EV_{M}^{\prime }\left(p_{2}\right)}{Q_{2}}\right)\left(1-\delta (1+A)\lambda \mu \frac{Q_{M}^{\prime }\left(m_{M2}\right)}{-dQ_{1}/dp_{1}}\right)$.

#### Proof.

See Appendix A.1. □

The condition reveals the key economic forces that drive declining prices under lagged reimbursement. The first term on the right-hand side of the inequality reflects how today’s price affects longer-run profitability beyond the immediate next period. It is at least 1 by the assumption that continuation profits are weakly increasing in the second period price. The second term captures how current pricing decisions propagate into future demand through reimbursement. To see the intuition underlying the condition, consider the case where profits after period 2 are not impacted by period 2 price, so $EV_{M}^{\prime }\left(p_{2}\right)=0$ and the first term is simply 1. Then, the condition reduces to $\frac{\eta_{2}}{\eta_{1}}$ being greater than something which is less than 1. It is thus always satisfied when the semi-elasticities with respect to margin are weakly larger in period 2. It is more likely to be satisfied when period 1 demand is relatively inelastic $\eta \left(m_{1}\right)<\eta \left(m_{2}\right)$, when Medicare is more generous (A is larger) and more important (μ is larger).^10^

To facilitate application of the theory to our empirical results, we have the following corollary:

#### Corollary 1.

*When* μ=0, *price is constant over time. When* μ>0, *price will decline over time as long as the conditions in Proposition 1 are satisfied.*

#### Proof.

When μ=0, it follows immediately from Eqs. (1) and (2) that $p_{1}=c+\frac{1}{\eta_{1}}$ and $p_{2}=c+\frac{1}{\eta_{2}}$. Since private reimbursement is constant over time, $\eta_{t}=\frac{-Q_{P}^{\prime }\left(\lambda \rho -p_{t}\right)}{Q_{P}\left(\lambda \rho -p_{t}\right)}$, and thus $p_{1}=p_{2}$. □

When μ=0, the theory tells us price is constant over time, since it is determined by the private insurance reimbursement rate, which is assumed to be constant in our model. The corollary is applicable, as Table 1 shows the average Medicare market share in the set of drugs below the median Medicare market share is close to zero– only 6%, compared to 48% in the above median group.

Finally, the theory has focused on how lagged-price reimbursement affects the slope of the price path over time, which is the focus of the empirical analysis. However, the *level* of the price path is important for Medicare’s overall spending. Intuitively, the initial Medicare reimbursement rate $r_{1M}$ affects the extent to which the pharmaceutical firm can set high prices in the initial period.

#### Proposition 2.

*The equilibrium prices in periods 1 and 2 are both increasing in the initial Medicare reimbursement*, $r_{1M}$.

#### Proof.

See Appendix A.1. □

Proposition 2 shows that the initial reimbursement rate set by Medicare shifts the level of the entire price path. The intuition is straightforward: the physician’s margin is determined by the change in price over time. If Medicare increases the initial reimbursement, initial and future prices can both increase, while maintaining the same margin for the physician. Future work examining how to optimally choose the initial level of reimbursement will likely be valuable.

These results show that the dependence of drug reimbursement on lagged prices can distort dynamic pricing decisions, leading firms to set high prices at launch and lower prices in subsequent periods. The mechanism is that margins, rather than prices per se, determine quantity: by raising launch prices and then reducing prices later, the firm generates higher physician margins in later periods. At the same time, Proposition 1 shows that lagged-price reimbursement makes comparative statics with respect to Medicare’s market share ambiguous: depending on how demand evolves and other parameters, lagged-price reimbursement can raise or lower prices over time. Given this theoretical ambiguity, the impact of this policy is an empirical question. We now turn to the data to examine how lagged-price reimbursement plays out in practice.

## 4. Data and descriptive statistics

### 4.1. Data

We construct a sample of prices and Medicare market shares for physician-administered drugs spanning 2006 through 2019. Our unit of analysis is the drug-quarter, where drugs are uniquely identified by Health Care Procedure Coding System (HCPCS) codes. We combine data from three sources: pricing files, aggregate Medicare claims, and Truven Marketscan spending aggregates.

To measure the price and reimbursement of a drug, we use the ASP of Part B drugs from 2005 through 2019, which the Centers for Medicare & Medicaid Services (CMS) make publicly available (CMS 2005–2019). ASP data are reported at the HCPCS level. We include only drugs introduced later than 2005 and exclude drugs in the ASP files that are reimbursed under alternative methodologies (vaccines and blood/clotting products).^11^ We use CMS Part B Summary file data dictionary code ranges to categorize these drugs into three categories: chemotherapy drugs, injections (J0120–J7175, e.g., aflibercept to treat age-related macular degeneration), and “other.”

We measure price by inverting the reimbursement rate. The ASP pricing files contain quarterly data on the reimbursement rate, which is a function of the lagged sale price of the drug. We construct our price variable for each drug j in quarter t by taking the reimbursement rate from quarter t+2 and dividing it by 1.06.^12^ ASP is measured per some standardized dosage (that is constant over time).

To measure exposure to the Medicare Part B program, we construct Medicare market share (MMS) for each HCPCS, following the reweighting approach recommended by Dunn et al. (2014) and used in Yurukoglu et al. (2017). We aggregate drug payments both from private insurers and from Medicare, and define MMS for each drug-year as Medicare over Medicare plus private drug payments in that year.

We obtain Medicare’s aggregate drug payments from the CMS Part B National Summary Data File, which contains yearly data on aggregate payments for each drug covered by Part B. We obtain aggregate private drug payments at the drug-year level using Truven MarketScan data for each year. We follow Yurukoglu et al. (2017) and scale these payments up by the ratio of all commercial insurance enrollees to the number of Marketscan enrollees in that particular year, assuming that Marketscan provides an approximately nationally representative sample of the commercial insurance market, which allows us to construct a national private drug payments figure.^13^

Our key treatment variable is a drug j’s Medicare market share at launch, which we term $MMS_{j}$. We focus on MMS at launch to measure a persistent characteristic of a drug– its exposure to the pricing incentives created by Medicare Part B. Finally, drugs are launched in different years, so we use τ to describe time in quarters relative to a drug’s launch. The first period that HCPCS is observed is normalized to be τ=1.

### 4.2. Descriptive statistics

**Fig. 2.**
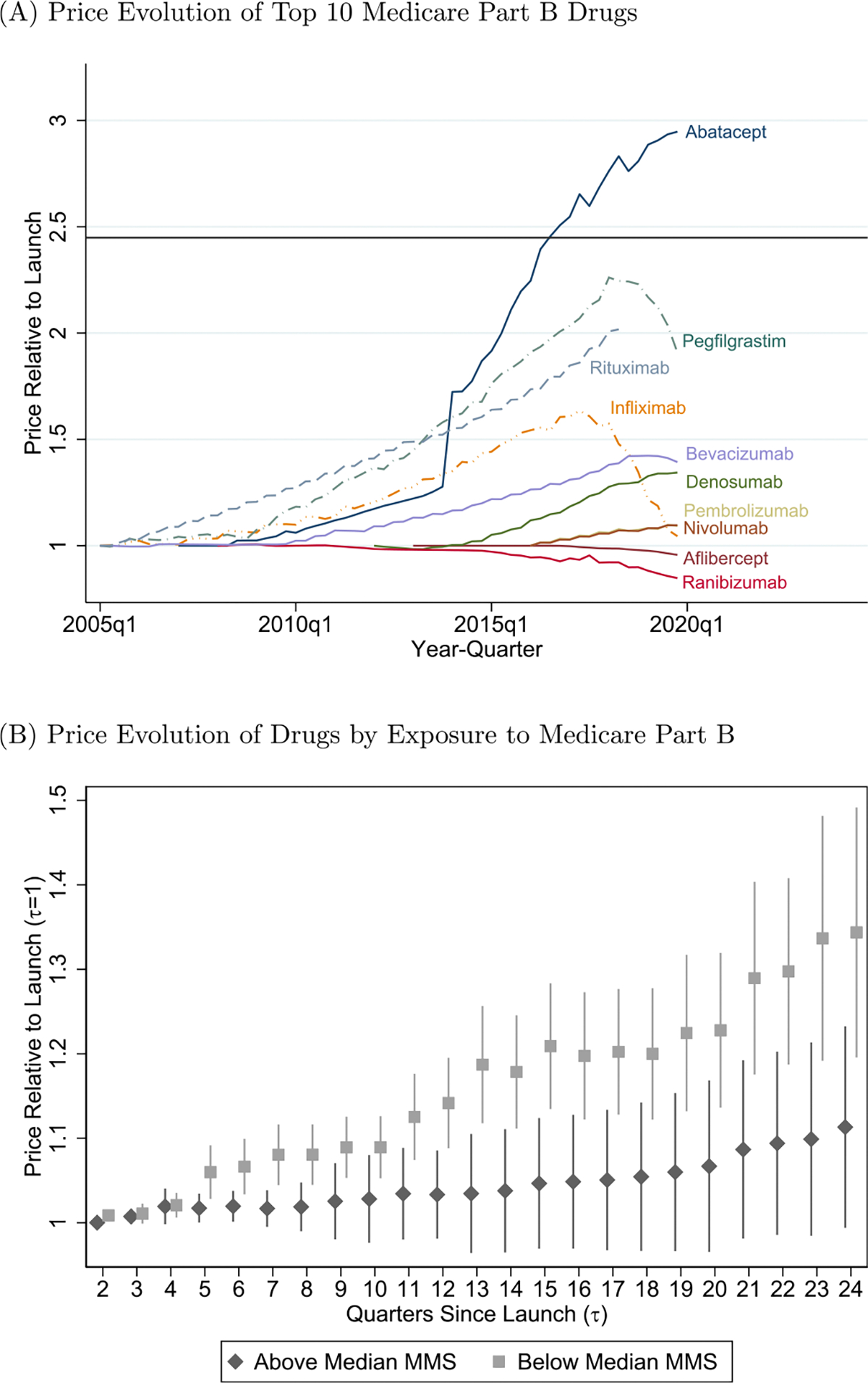
Price Evolution of Medicare Part B Drugs. *Notes:* Panel A: Selects the top 10 Part B drugs by Medicare revenue 2015–2019. Panel B: Price relative to launch by exposure to Medicare. Relative price is ASP in quarter τ divided by ASP in quarter τ=1. Plots the results of a regression of relative quantity against quarter τ fixed effects and year fixed effects weighted by total drug market size.

Prices evolve quite heterogeneously across drugs. We give some examples in Fig. 2 Panel A, which displays the price paths (relative to launch price) of the top 10 Medicare Part B expenditure drugs across the 2005–2019 period. We plot prices relative to prices in 2005, though we note that these drugs were introduced at a variety of different times. While many of these drugs show a steady increase across time, there are exceptions, such as Ranibizumab (a drug used for macular degeneration), that show declining prices over time. Various drugs experience a drop in prices after prior increases; these drop-offs are sometimes but not always related to billing-code entry.

Medicare quantity sold, measured as revenue divided by price, varies over time (see Appendix Figure A2). While highly heterogeneous across drugs, on average, quantity sold doubles in the first two years post-launch. As a result, prices in later periods contribute more to the volume-weighted lifetime cost of a drug than the launch price, motivating our analysis of the dynamic pricing impact of Part B reimbursement.

Table 1 provides descriptive statistics on our cohort of drugs, and Appendix Figure A3 illustrates the distribution of MMS at launch. We identify 215 unique HCPCS, and divide them into above and below median MMS at launch, which is 0.193. Relative prices two years after launch are about 3% higher for above median MMS drugs and 8% higher for below median MMS drugs. There are differences between the above and below median MMS drugs. Above median MMS drugs are about 1.3 years “newer” than below median drugs, and are more likely to be injections than chemotherapy or other drugs. We account for these differences in our empirical strategy by including controls for calendar time, as well as by running our regressions within drug-type subsamples.

Prices grow at a slow enough rate to leave positive profit margins for the average provider. Constructing the average annual and quarterly growth rate for each drug provides insight into the profits that prescribing providers make. A provider who acquires the drug at a price equal to ASP every quarter will make zero profit margin on a drug whose price grows at 6% over two quarters. (The reimbursement rate will equal acquisition costs in this case.) Note that for prices to grow by 6% over two quarters, the compound quarterly growth rate has to be 2.96%, since $(1.0296{)}^{2}=1.06$. Here, Table 1 shows that the mean compound quarterly growth rate is 0.92%.

Fig. 2 Panel B shows the price evolution of drugs over time, split by exposure to Part B. We split the sample by whether the drug was above or below median MMS at launch to allow us to compare prices between drugs that are more or less exposed to the Medicare market. This figure normalizes the price at launch (τ=1) and plots price in later quarters, weighting by total market size. Weighted by market size, drugs with above median exposure to Part B are about 30% more expensive six years after launch, but drugs below median exposure are only about 10% more expensive.

## 5. Empirical strategy and results

### 5.1. Estimation

Our empirical strategy uses cross-sectional variation in individual drug exposure to the Medicare market to identify the effects of Part B’s lagged ASP reimbursement rule on drug price growth. In our event study graphs, we estimate:

(3) $$ln\left(p_{jt}\right)=\alpha_{\tau_{jt}}MMS_{j}\times \tau_{jt}+\beta_{\tau_{jt}}\tau_{jt}+\theta X_{jt}+\epsilon_{jt}$$

where $p_{jt}$ is the price (ASP) of drug j in year t, and $MMS_{j}$ is the Part B share of drug j’s claims in its first quarter. We include $\tau_{jt}$, a set of indicator variables for the quarter relative to when drug j was introduced, multiplied by coefficients $\beta_{\tau_{jt}}$. We include both drug and year fixed effects in $X_{jt}$. Note that $MMS_{j}$ does not vary over time (it is constant within a drug).^14^ In other specifications we include quarters since launch linearly or as an indicator for more than 12 quarters since launch.

The key coefficients of interest are the $\alpha_{\tau_{jt}}$, the coefficients on the interactions between the time since launch indicators $\tau_{jt}$ and Medicare market share at launch $MMS_{j}$. The estimates describe how prices in later periods compare to the launch price for HCPCS with relatively high Medicare market share at launch. The identifying assumption is that drugs with different $MMS_{j}$ would have had the same percentage change in price in later periods in the absence of incentives created by the Medicare reimbursement program. Conditional on drug fixed effects, the regression coefficients can be interpreted as percentage price changes relative to launch. While we cannot identify the effect of Medicare market share on launch prices, this strategy has a number of advantages. For example, we do not need the price per standardized dosage of one drug to be comparable to the price per dosage of another.

We weight our regressions by a drug’s average total market size over the time of our sample. This allows us to identify the average causal effect of Medicare market share per dollar spent, rather than the average per drug, as there are many small drugs that are relatively unimportant for overall drug spending. (See Solon et al. (2015) on weights).

### 5.2. Main results

**Fig. 3.**
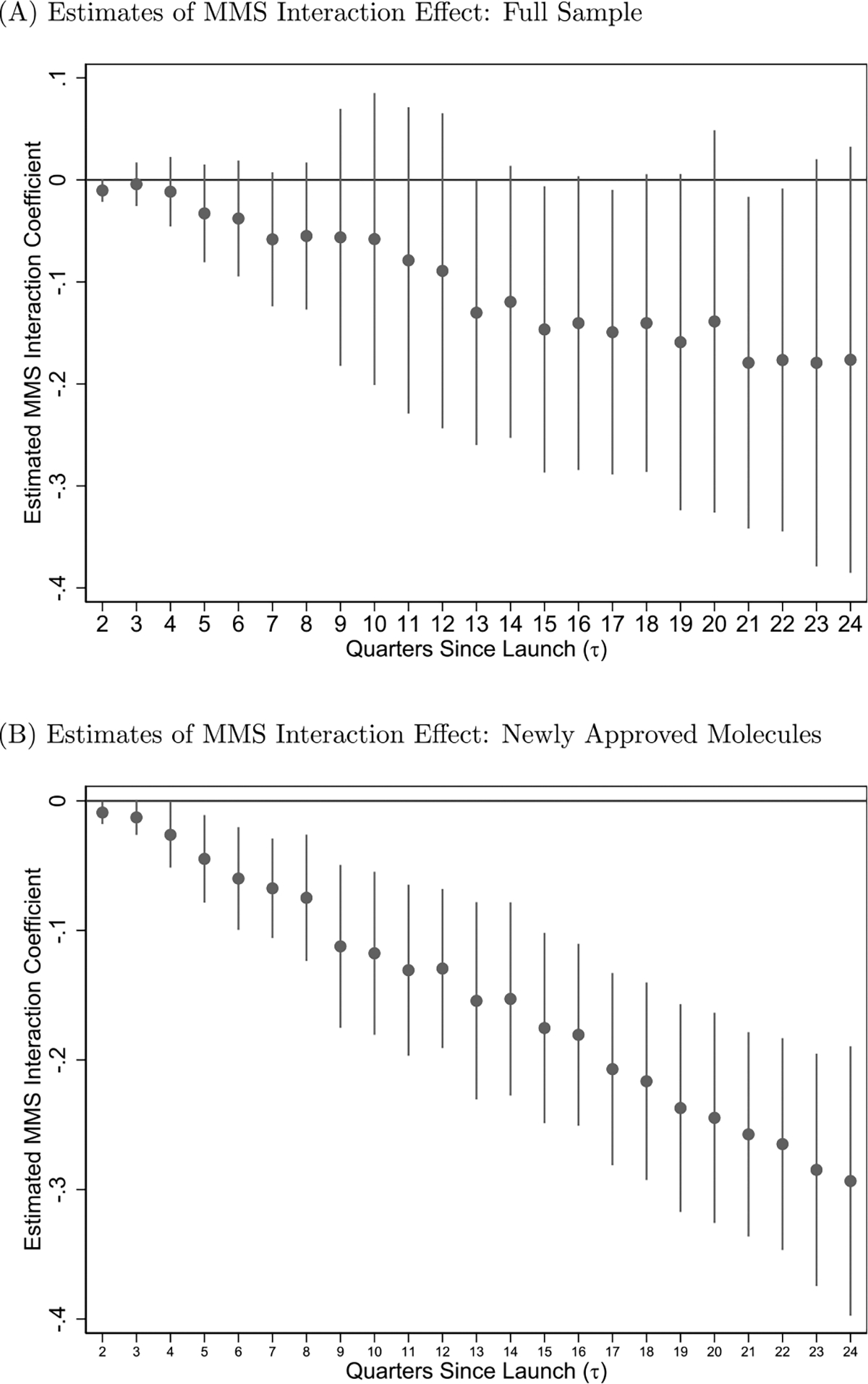
Exposure to Medicare Part B and Drug Prices. *Notes:* Panel A: Plots point estimates and 95% confidence intervals for coefficients estimated by the regression given in Eq. (3) on the Analysis Sample weighted by total drug market size. Panel B: Same as panel A, but estimated on sample of newly approved molecules. Robust standard errors clustered at the HCPCS level.

Panel A of Fig. 3 shows that drugs that are more exposed to Medicare Part B have slower price growth. The confidence intervals on each individual interaction coefficient are wide, but we can test the hypothesis that the interactions between MMS and time since launch are all zero (F(23,214)=3.52,p<0.001). We thus reject the hypothesis that high Medicare market share drugs have the same price path as low Medicare market share drugs.

To interpret the results, note that the interaction coefficient on $\tau \times MMS_{j}$ tells us how much higher drugs with 100% Medicare market share will be priced in period τ, relative to their launch price. The interaction coefficient of −0.18 on $\tau =24\times MMS_{j}$ tells us that high Medicare market share drugs will have increased their prices less than low Medicare market share drugs. For a drug sold only to Medicare ($MMS_{j}=1$), the estimates predict that after 6 years, its price would be 18 log points (16.4%) below a drug with no Medicare market share that launched at the same price. (They may, however, launch at different prices.)

We summarize our results succinctly in Table 2, which presents results for both the full analysis sample and a balanced panel of drugs. In Column 1, we impose a linear time trend in prices post launch, and interact that with MMS. These specifications indicate that the prices of drugs with zero MMS grow at about 0.7% per quarter, while drugs with 100% MMS grow about −0.8 percentage points less per quarter than zero MMS drugs. Results in Column 3 for a balanced panel show a similar pattern, but with a stronger interaction where MMS=1 drugs grow about −1.5 percentage points per quarter less than MMS=0 drugs.

However, a linear specification in time and MMS may not be appropriate.^15^ Table 2 also presents a specification in which time period is split into early (τ<=12) and late, and drugs are split into above and below median MMS. Holding launch price constant, prices of above median MMS drugs are 11% below those of below median MMS drugs after 3 years in our analysis sample, with a smaller estimate for the balanced panel.

Our main analysis examines price and reimbursement for all newly introduced J-codes, regardless of whether the underlying molecule was newly approved. However, the pricing dynamics of older drugs might be different, due to greater generic competition (or the threat thereof), as well as potential anchoring on prices that pre-dated the introduction of the new HCPCS code. Indeed, Appendix Figure A4 shows that competitor entry can happen quickly for new J-codes for existing molecules.

To address these concerns, we construct a sample of drugs that are less exposed to competition. We created a narrow sample of drugs whose molecule FDA approval date was concurrent (within 1 year) with the introduction of the HCPCS code.^16^ The resulting estimates displayed in Panel B of Fig. 3 show that the MMS interaction effects are larger in magnitude and more precisely estimated in this sample, with coefficients of approximately −0.13 after 12 quarters and −0.29 after 24 quarters, compared to about −.09 and −0.18 in our main results. This suggests our main specification is conservative.^17^

### 5.3. Robustness

Appendix Figure A6 shows that our results are robust to using a variety of alternative two-way fixed effects estimators. Point estimates in each case are quite similar. (To test robustness to alternative estimators, we need to discretize our treatment. We do this by splitting our sample into above versus below median MMS, as in Table 2.)

We also consider a series of additional robustness checks in Appendix Figure A7. Each panel presents an analogue of Fig. 3 Panel A run on a different sample. In Panel A, we show that excluding outliers does not meaningfully affect our results. In Panel B, we weight all drugs equally, rather than by drug market size. The results are noisier, though the point estimates are larger in magnitude. To address any concerns that our results are driven by an unbalanced panel, we construct a sample with a balanced panel. We first shorten our estimation window to the first 4 years since launch in order to maximize sample size, and show the regression results on an unbalanced panel in Panel C. Panel D then shows the balanced panel results. The results are quite similar, and in fact more precisely estimated than our main results.

In Appendix Figure A8, we consider a set of additional robustness checks related to sample composition. Panel A reproduces our main Fig. 3 on a common axis for reference. The negative trend becomes, if anything, stronger in three robustness checks: excluding the initial (and largest) cohort of observations in Panel B, excluding small cohorts of two or fewer drugs in Panel C, and excluding drugs that ever have a period of missing price data in Panel D. We also examine whether the effect of MMS is different in cohorts of drugs introduced sooner versus later after Part B’s lagged-price reimbursement system was introduced. Appendix Table A1 shows no clear evidence that the effect is different for these cohorts.

To address concerns that pricing might vary differentially by MMS due to differences in types of drugs, rather than the reimbursement incentives, we separately consider broad classes of products (chemotherapy, injections, or other). Appendix Table A2 shows the results. These smaller subsamples have less precision. For injections, we find significant results in line with our main results. The results for the smaller categories (chemotherapy and the “other” category) are much less precise, and confidence intervals include both zero effects and large negative effects like those found in our main results.

Finally, to address concerns that MMS might be endogenous to firm pricing strategy, we create another independent measure of exposure to Medicare’s pricing incentives. We examine individuals with commercial insurance not on Medicare, and compare drugs with higher market share among older versus younger commercially insured individuals. While this measure will not account for potential spillovers across markets (as in Dean et al. (2023)), it removes any variation due to strategic pricing by manufacturers. The correlation at launch between MMS and this alternative measure is 0.46. Appendix Tables A3 and A4 provide details and show that we estimate similar and perhaps larger in magnitude negative impacts of this alternative measure of exposure to Part B’s incentives on price growth.

### 5.4. Medicare market share and launch price

To place in context our estimates of the dynamic effects of lagged-price reimbursement, we also provide estimates of the impact of Medicare Part B on initial launch price. We view these estimates with skepticism. Our main results include drug fixed effects and simply require that counterfactual percentage changes in prices be the same across groups. However, to identify whether drugs with higher Part B exposure have higher launch prices, we must remove drug fixed effects from our regression. The identification assumption required is now much stronger: in the absence of Part B’s reimbursement formula, the types of drugs with greater exposure to Medicare Part B would have had initial prices that were the same on average as drugs with less exposure to Part B. Moreover, in the absence of a clearly comparable unit of measure for drug pricing, we anticipate greater variation in measured HCPCS prices.

Nonetheless, Table 3 shows the results of regressions that parallel those in our Table 2, but now drop drug fixed effects and display the effect of MMS on launch prices. We estimate that drugs with above median MMS have launch prices that are 64 log points (89%) higher, but this is imprecisely estimated, and we cannot reject declines of 25 log points or increases of 152 log points. Despite the imprecision, these results plus those of Ridley and Lee (2020), suggest that the effects of Part B on initial price are larger than the declines in later periods.^18^

### 5.5. Implications

What is the overall impact of high (above-median) exposure to Medicare on the lifecycle price of drugs? We compare our estimates to the estimates we would get if we ignored the dynamic price effect and simply extrapolated the estimated Part B effect on launch price to all future periods. We focus on the first 6 years (24 quarters), and assume no difference after that time, as this is the window for which we are able to estimate results. The lifecycle price of a drug is $\frac{\sum_{\tau =1}^{24}p_{\tau }Q_{\tau }}{\sum_{\tau =1}^{24}Q_{\tau }}$, ignoring discounting over this short horizon.^19^ Each period’s price is weighted by the quantity sold $Q_{\tau }$ in that period using the estimates from Figure A2.

Our estimate of the effect of being above median MMS in years 3–6 comes from Table 2 Column 2. The estimate of the effect of being above median MMS on launch is taken from Table 3 Column 2. We transform these log point changes into percentage changes. Naively extrapolating the launch price effect implies that being above median MMS raises lifecycle price by 89%. However, the lifecycle price is actually only 79% higher accounting for the decline in years 3–6. Ignoring the dynamic price changes would lead to an overestimate of the lifecycle price.

The estimated net effect is that more exposure to Part B leads to higher lifecycle prices. This result is robust to a range of possible launch price effects– it holds even if the actual increase in launch price was only about one-tenth of our observed estimate.^20^

Finally, our results can be an underestimate of the impact of Medicare’s reimbursement policy if commercial insurers follow Medicare, as some previous research has suggested (e.g., Clemens and Gottlieb (2017)). Indeed, our strategy should estimate no effect if all commercial insurers exactly followed Medicare.

## 6. Conclusion

Understanding the dynamic pricing incentives in lagged-price reimbursement contracts is important. Our model and empirical analysis show that these contracts can shape the market prices that they in turn rely on. We find that lagged-price cost-plus reimbursement in Medicare Part B creates incentives to launch at high prices and then lower them over time to give provider’s more margin on their purchases. The dynamic effects of the contract thus dampen the upward pressure on prices generated by the Medicare program. Moreover, our identification strategy may underestimate the effect of Medicare Part B’s reimbursement scheme, to the extent that private payers follow Medicare.

Future theoretical work could explore the impact of price dispersion and negotiation in the model. Future empirical work could examine how providers respond to changes in margin and how that affects patient health. Moreover, because Medicare Part B reimbursement design affects prices and thus drug profitability, it may have impacted innovation. Looking outside the US, empirical work could examine the dynamic pricing incentives of external reference pricing in Europe, as these reimbursement systems also rely on lagged prices.

Understanding how payment policy affects the pricing of pharmaceuticals is necessary to evaluate policy reforms, such as the US’s drug price negotiation reforms included in the 2022 Inflation Reduction Act. The impact depends on both policy parameters and the elasticity of demand. Our model can be used by policy-makers with context-specific estimates to predict how the design of contracting in Medicare Part B will impact overall costs. It could also enrich models of external reference pricing (in which countries set prices based on lagged prices in other countries; see e.g., Maini and Pammolli (2023)) and can be used for other non-pharmaceutical industries.

## Supplementary Material

MMC1

## Figures and Tables

**Table 1:** Descriptive statistics of drugs.

	Full Sample		Above Median MMS	Below Median MMS
			
	*Mean*	*Std Dev*	*Mean*	*Mean*

Medicare Market Share (MMS) at Launch (*τ* = 1)	0.270	0.261	0.480	0.062
Relative ASP at 8 Quarters Since Launch (*τ* = 8)	1.058	0.335	1.032	1.083
Average Year of Introduction	2010.7	3.8	2011.4	2010.1
Compound Annual Growth Rate over first 6 years	0.0409	0.1000	0.0196	0.0572
Compound Quarterly Growth Rate over first 6 years	0.0092	0.0239	0.0041	0.0131
N (Unique HCPCS)	215		107	108
*Shares By Drug Category:*				
Chemotherapy	23%		29%	17%
Injections	60%		42%	77%
Other	18%		29%	6%

Source: Authors’ calculations from CMS Data 2006–2019. Median Medicare Market Share at Launch = 0.193. Percentages may not add to 100% due to rounding.

**Table 2:** Summarizing the Effect of MMS on Price Evolution.

	(1)	(2)	(3)	(4)
	
	Analysis Sample		Balanced Panel	

Quarters Since Launch (*τ*)	0.007 (0.005)		0.008*** (0.002)	
MMS × Quarters Since Launch (*τ*)	−0.008* (0.005)		−0.015*** (0.005)	
Quarters Since Launch (*τ*) > 12		0.079** (0.035)		0.033** (0.014)
Above Median MMS × Quarters Since Launch (*τ*) > 12		−0.111** (0.051)		−0.030*** (0.005)
Drug Fixed Effects	Yes	Yes	Yes	Yes
Year Fixed Effects	Yes	Yes	Yes	Yes
*R* ^2^	0.070	0.087	0.383	0.364
N	4502	4502	588	588

Notes:

*** *p* < 0.01

** *p* < 0.05

* *p* < 0.1

Dependent variable: ln *p_jt_*. Robust standard errors clustered at the HCPCS level. Balanced panel only uses observations with *τ* ≤ 16 and requires that all drugs have at least *τ* = 16.

**Table 3:** Estimates of the Effect of MMS on Launch Price.

	(1)	(2)	(3)	(4)
		
	Weighted		Unweighted	

MMS	2.650*** (0.875)		1.022 (0.730)	
Above Median MMS		0.639 (0.449)		0.503 (0.349)
Drug FE	No	No	No	No
Year FE	Yes	Yes	Yes	Yes
*τ* FE	Yes	Yes	Yes	Yes
*τ* FE × MMS	Yes	No	Yes	No
*τ* FE × Above Median MMS	No	Yes	No	Yes
R-squared	0.220	0.060	0.020	0.020
N	4502	4502	4502	4502

Notes:

*** *p* < 0.01

** *p* < 0.05

* *p* < 0.1.

Dependent variable: ln *p_jt_*. Data: Analysis Sample. Robust standard errors clustered at the HCPCS level.

## Data Availability

Data will be made available on request.

## HIGHLIGHTS

- Lagged Medicare reimbursement affects pharmaceutical price trajectories.
- Physician-administered drugs with higher exposure to Medicare experience slower price growth.
- Price dampening emerges several years after market entry.
- Effects are strongest for new drugs facing limited early competition.
- Results inform the design of cost-plus procurement contracts.

## Footnotes

1 See economic price adjustment clauses in construction and defense contracts discussed in Crocker and Reynolds (1993) and Kosmopoulou and Zhou (2014).

2 In addition to the 2022 Inflation Reduction Act, e.g., as discussed in Cutler (2022), potential policy reforms are discussed in Ridley and Zhang (2017); Dubois et al. (2022); Ginsburg and Lieberman (2021). Lakdawalla (2018) provides a review of the literature on the economics of pharmaceuticals. By contrast, in Medicare Part D, which covers most self-administered drugs, the government has largely devolved price negotiation to private firms that offer insurance plans.

3 Duggan and Scott Morton (2010) also use this strategy to show that the introduction of Medicare Part D *lowered* the cost of covered drugs, as plan formularies made the demand of newly insured individuals more elastic. Ippolito and Levy (2023) also show that drugs more exposed to Medicare Part D have larger differences between net and list prices of drugs.

4 The controversial 340B program allows some providers treating low-income patients to buy at a discount and (in some time periods) receive lower reimbursement. See Desai and McWilliams (2018).

5 Patient cost-sharing is substantially reduced if they purchase additional Medigap insurance or are dual-eligible for Medicaid.

6 This can be generalized to multiple periods but two periods suffice to show the dynamics.

7 While this assumption is stylized, it tractably captures the dynamic incentives introduced by the Medicare reimbursement contract. In practice, acquisition prices depend on a variety of supply-side factors, such as negotiations between providers and drug suppliers (Medicare Payment Advisory Commission, 2016b). Yet it is only through the *average* price that drug suppliers can affect the future Medicare reimbursement rate.

8 Allowing patient utility to enter the physician utility function with some weight is a common way to proxy for the result of a joint patient-physician decision (McGuire, 2000).

9 Cost-sharing depends on insurer reimbursement, *not* physician acquisition price. This cost-sharing can vary across patients depending on whether they have supplemental coverage, but we abstract away from that in the theory.

10 Although the model has just two periods, the continuation value can be reinterpreted as a summary of the effect of price on discounted profits over any number of future periods. Applying the result to a multi-period setting, a larger $EV_{M}^{\prime }\left(p_{2}\right)$– indicating stronger dynamic incentives– narrows the range of parameters consistent with falling prices but does not alter the qualitative mechanism.

11 That is, we limit our analysis to the set of HCPCS codes that begin with “J”, which corresponds precisely to physician administered drugs reimbursed under the ASP methodology Centers for Medicare & Medicaid Services (CMS) (2006–2019).

12 The files report the Medicare payment limit, which corresponds to 80% of the statutory reimbursement rate, so there is an additional re-scaling step necessary. The reimbursement rate in these files is the statutory “allowed amount” for the drug, which remains 106% of ASP throughout the sample period. The Budget Sequester Act in 2013 effectively reduced the Medicare component of the payment but the statutory rate remained at 106%.

13 We estimate the number of commercial insurance enrollees to be 295 million (total number of insured persons in the US) minus 89 million (total number of Medicare enrollees), which is equivalent to the Ridley and Lee (2020) re-scaling.

14 MMS is highly correlated within a drug over time, above 0.9.

15 While Fig. 3 Panel B shows a linear decline in quarters since launch, that pattern is less clear in Panel A. Moreover, the effect of MMS itself need not be linear.

16 This required merging the HCPCS to NDC using string matching on drug names. The narrow sample is smaller– 88 unique HCPCS, rather than 215 in our main analysis sample. Appendix Figure A5 shows that this sample has a longer time until competitor entry in the billing code.

17 A linear specification in time gives a coefficient on τ×MMS of −0.013, about twice that in Table 2 column 1.

18 Ridley and Lee (2020) estimates that the average effect of being exposed to Part B’s lagged-price reimbursement payment system, compared to Medicare’s previous reimbursement system, was a 0.61 log point price increase at launch. That estimate is not directly comparable to ours, as our estimate comes from variation in exposure to Medicare versus private payment.

19 The results below round to the same percentage change for annual discount rates up through 6 percent.

20 Pharmaceutical firms may discount future profits at a higher rate than government agencies or society, which can affect the optimal design of reimbursement mechanisms. Interestingly, both the reimbursement system we study and patent protection regimes front-load profits.

## Appendix A. Supplementary data

Supplementary data to this article can be found online at doi: 10.1016/j.jpubeco.2026.105595.
