# Supplementary material for: Lagged-price reimbursement contracts: The impact of medicare Part B on pharmaceutical price growth
Source: J Public Econ. Author manuscript; Available in PMC 2026 Jul 21. (PMC13384466; doi:10.1016/j.jpubeco.2026.105595)
Supplement: MMC1 [file NIHMS2151295-supplement-MMC1.zip › jpubec_appendix_V3.pdf]

Lagged-Price Reimbursement Contracts: The Impact of Medicare Part B on Pharmaceutical Price Growth

Angelique Acquatella, Keith Marzilli Ericson, and Amanda Starc*

February 12, 2026

Online Appendix For: "Lagged-Price Reimbursement Contracts: The Impact of Medicare Part B on Pharmaceutical Price Growth"

Angie Acquatella, Keith Marzilli Ericson, and Amanda Starc

A Theoretical Appendix

Assumptions

Formally, the assumptions we make about the demand system are:

(i) effective margin is increasing in reimbursement ($\lambda > 0$), and the patient out-of-pocket share is constant, $\frac{oop(r_t)}{r_t} = k \; \forall r_t$ for some $k \in \mathbb{R}^+$;

(ii) demand is weakly concave in the effective margin $Q''(m_t) \leq 0$;

(iii) the effect of p_2 on continuation profits is non-convex, such that $EV''_M(p_2) \leq 0$.

*Acquatella: Toulouse School of Economics. angelique.acquatella@tse-fr.eu. Ericson: Questrom School of Business, Boston University, and NBER. kericson@bu.edu. Starc: Kellogg School of Management, Northwestern University. amanda.starc@kellogg.northwestern.edu. The authors gratefully acknowledge research support by grants from the NIHCM and NIA grant P30AG012810. We thank Mohan Ramanujan and Elizabeth Adams for their assistance in obtaining and managing the data. We thank Taylor Watson for excellent research assistance. We thank Rena Conti, David Ridley, and seminar participants at Boston University, Indiana University, ASHEcon, and the Highland Health Economics Symposium for helpful comments.

(iv) cross-effect bound: Let F_1 and F_2 be the first-order conditions with respect to p_1 and p_2, respectively. Let the Hessian be $H = \begin{bmatrix} \frac{\partial F_1}{\partial p_1} & \frac{\partial F_1}{\partial p_2} \\ \frac{\partial F_2}{\partial p_1} & \frac{\partial F_2}{\partial p_2} \end{bmatrix}$. To guarantee the second order conditions hold, we require that $\det(H) = \frac{\partial F_1}{\partial p_1} \cdot \frac{\partial F_2}{\partial p_2} - \left(\frac{\partial F_1}{\partial p_2}\right)^2 \geq 0$

This requires the cross effect on the right-hand side of the below equation not be too large:

$$\frac{\partial F_1}{\partial p_1} \cdot \frac{\partial F_2}{\partial p_2} \geq \delta^2 \mu^2 \lambda^2 (1+A)^2 \Big[Q'_M(M_2) - (p_2 - c)\, Q''_M(M_2) \Big]^2. \tag{A.1}$$

Assumptions ii-iv guarantee that the second-order conditions hold.

A.1 Proofs

Proof of Proposition 1. Based on the definition of η_2, we can rewrite the FOC for period two price as $p_2 = c + \frac{1}{\eta_2}\left(1 + \frac{\delta \mu EV'_M(p_2)}{Q_2}\right)$. This expression for $(p_2 - c)$ can be substituted into the FOC for period 1 price. Then, the difference between the optimal prices in period 2 and period 1 from equations (1) and (2) can be written as:

$$p_1 - p_2 = \frac{1}{\eta_1} - \frac{1}{\eta_2}\left(1 + \frac{\delta \mu EV'_M(p_2)}{Q_2}\right)\left(1 - \frac{\delta \mu \lambda (1+A) \cdot Q'_M(m_{M2})}{-dQ_1/dp_1}\right) \tag{A.2}$$

Given that $\eta_1 > 0$ and $\eta_2 > 0$, it follows directly from equation A.2 that $p_1 - p_2 > 0$ if and only if: $\frac{\eta_2}{\eta_1} > \left(1 - \frac{\delta \lambda (1+A) \mu \cdot Q'_M(m_{M2})}{-dQ_1/dp_1}\right)\left(1 + \frac{\delta \mu EV'_M(p_2)}{Q_2}\right)$. Notice that $\left(1 + \delta \mu \frac{EV'_M(p_2)}{Q_2}\right) \geq 0$ because $EV'(p_2) \geq 0$ by assumption. However, the sign of $\left(1 - \frac{\delta \lambda (1+A) \mu \cdot Q'_M(m_{M2})}{-dQ_1/dp_1}\right)$ is ambiguous. ■

Proof of Proposition 2. The optimal prices (p_1, p_2) satisfy the first order conditions stated in Equations 1 and 2. For ease of exposition, we rearrange the first-order conditions and write them as: $F_1(p_1, p_2, r_{M1}) \equiv Q_1 - (p_1 - c)\left[\mu Q'_M(m_{M1}) + (1-\mu) Q'_P(m_{P1})\right] + \delta(p_2 - c)\mu\lambda(1+A) Q'_M(m_{M2}) = 0$ and $F_2(p_1, p_2) \equiv \delta(Q_2 - (p_2 - c)[\mu Q'_M(m_{M2}) + (1-\mu) Q'_P(m_{P2})]) + \delta^2 \mu EV'_M(p_2) = 0$. Totally differentiating with respect to r_{M1} yields the system:

$$\begin{bmatrix} \frac{\partial F_1}{\partial p_1} & \frac{\partial F_1}{\partial p_2} \\ \frac{\partial F_2}{\partial p_1} & \frac{\partial F_2}{\partial p_2} \end{bmatrix} \begin{bmatrix} \frac{dp_1}{dr_{M1}} \\ \frac{dp_2}{dr_{M1}} \end{bmatrix} = \begin{bmatrix} -\frac{\partial F_1}{\partial r_{M1}} \\ 0 \end{bmatrix} \tag{A.3}$$

By Cramer's rule, the solution to this linear system is given by the ratio of determinants, where each numerator is obtained by replacing the corresponding column of the coefficient

matrix with the right-hand side vector:

$$\frac{dp_1}{dr_{M1}} = \frac{-\frac{\partial F_1}{\partial r_{M1}} \cdot \frac{\partial F_2}{\partial p_2}}{\det(H)}, \quad \frac{dp_2}{dr_{M1}} = \frac{\frac{\partial F_1}{\partial r_{M1}} \cdot \frac{\partial F_2}{\partial p_1}}{\det(H)} \tag{A.4}$$

where $\det(H) = \frac{\partial F_1}{\partial p_1} \cdot \frac{\partial F_2}{\partial p_2} - \frac{\partial F_1}{\partial p_2} \cdot \frac{\partial F_2}{\partial p_1}$ is the determinant of the Hessian. Since (p_1, p_2) maximize profits, the second-order conditions require H to be negative definite, which implies $\det(H) > 0$. Further, $\frac{\partial F_2}{\partial p_2} < 0$ by concavity.

To establish the sign of $\frac{\partial F_1}{\partial r_{M1}}$, note that:

$$\frac{\partial F_1}{\partial r_{M1}} = \lambda\mu \left[Q'_M(m_{M1}) - (p_1 - c)Q''_M(m_{M1})\right] \tag{A.5}$$

Under the assumption that demand is concave in the effective margin, we have $\frac{\partial F_1}{\partial r_{M1}} > 0$ since $\lambda, \mu > 0$ and $Q'_M \geq 0$. Similarly, $\frac{\partial F_2}{\partial p_1} = \delta(1 + A)\lambda\mu[Q'_M(m_{M2}) - (p_2 - c)Q''_M(m_{M2})] > 0$ by the same argument. Therefore, both numerators are positive, establishing $\frac{dp_1}{dr_{M1}} > 0$ and $\frac{dp_2}{dr_{M1}} > 0$.

■

B Extensions

B.1 Simulations with More Periods

To illustrate how the pricing dynamics generalize beyond two periods, we conducted numerical simulations for a ten-period horizon ($T = 10$). Just as in our main text's simulations, the price dynamics will depend on the parameters.

Appendix Figure A1 shows the results for two different parameter sets. Each figure plots optimal prices across periods for three values of Medicare exposure: $\mu = 0$ (no Medicare market), $\mu = 0.25$, and $\mu = 0.5$. The $\mu = 0$ line is flat because, in the absence of Medicare, there is no dynamic link between periods. Comparing $\mu = 0.25$ and $\mu = 0.5$ shows how greater Medicare exposure amplifies the dynamic pricing pattern.

In Case 1, prices decline gradually in early periods and more steeply near the end of exclusivity. This is similar to the empirical pattern of monotonic decline we see in the paper's Figure 3.

In Case 2, the profit-maximizing firm prefers not to launch at a very high initial price relative to first-period reimbursement. Instead, as lagged reimbursement increases, the firm gradually raises prices before reducing them in later periods.

With more periods, pricing dynamics can be more complex than in a two period model. However, these simulations show similar pricing motivations: higher average prices early in the product's life to invest in Medicare reimbursement, followed by lower prices later to harvest quantity.

Figure A1: Pricing Dynamics With a Longer Time Horizon

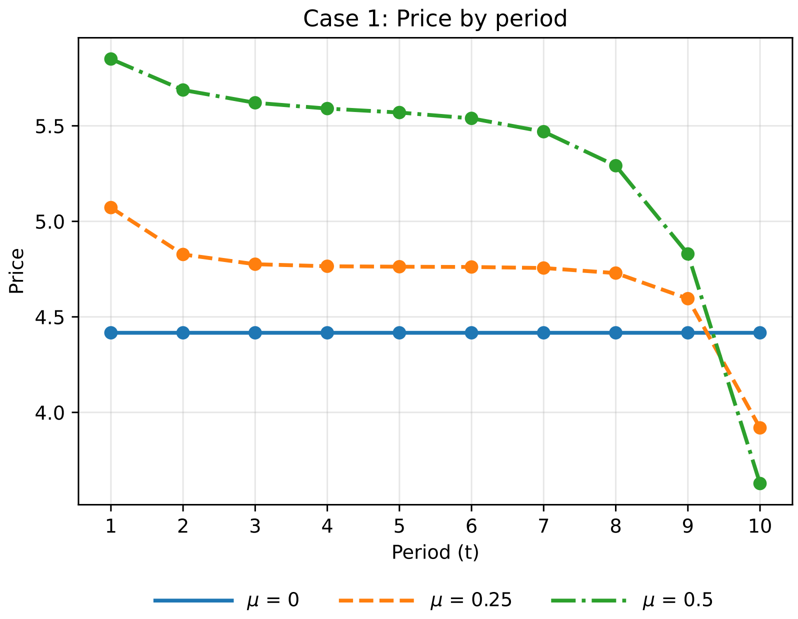

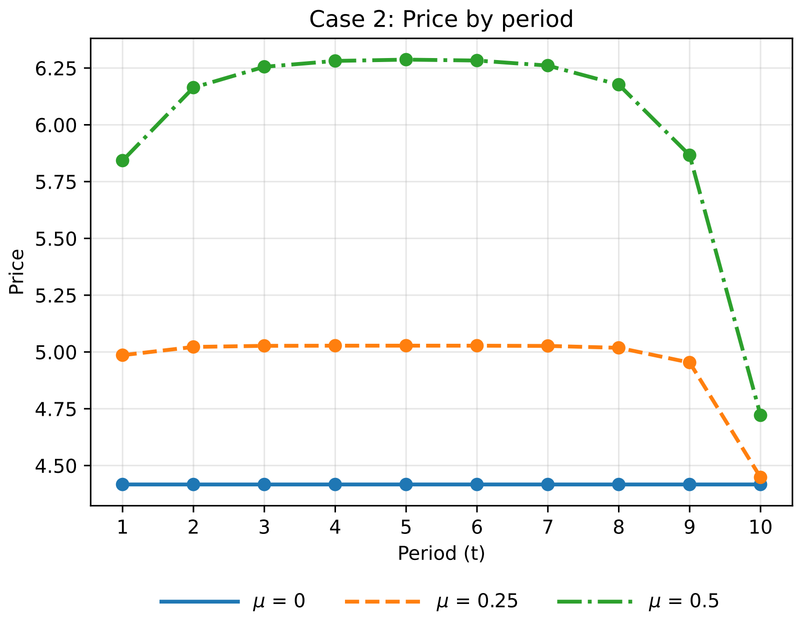

Notes: Authors' Simulations. In both cases, unit cost $c = 0.5$, discount factor $\delta = 0.95$, $\lambda = 1.0$, private reimbursement $\rho = 5.0$, and continuation value after $T = 10$ is zero. Case 1: Has a large lagged price markup $A = 0.12$, high initial Medicare reimbursement: $r_{M,1} = 7.0$. Demand asymmetric across payers: Medicare demand has a low intercept of $(a_M = 0.08)$ but a steep slope of $(b_M = 0.20)$, while private demand has a higher intercept of $(a_P = 0.40)$ and less steep slope of $b_P = 0.12$. Case 2: Has a lower lagged price markup $A = 0.06$, and initial Medicare reimbursement that matches private reimbursement $r_{M,1} = 5.0$. Demand is symmetric across payers, with intercept $a_M = a_P = 0.40$ and slope $b_M = b_P = 0.12$.

B.2 Distinct Drug Pricing Across Medicare versus Privately Insured Patients

Suppose instead that the provider faces different acquisition prices depending on whether they have a Medicare patient (p_t^M) or a privately insured patient (p_t^P). This case may be of interest, as evidence suggests there is substantial heterogeneity in acquisition price across providers (Medicare Payment Advisory Commission 2016b), and a monopolist seller can price-discriminate. Medicare's lagged price reimbursement is now $r_t = (1+A)(\mu p_{t-1}^M + (1-\mu)p_{t-1}^P)$. All other ingredients of the model remain the same.

The physician's effective margin in period t is now given by: $m_{it} \equiv \lambda r_{it} - p_{it}$, where $i = \{M, P\}$. Given $\mathbf{r} = \{r_1^M, r_2^M(p_1^M), \rho\}$, the pharmaceutical firm chooses a vector of Medicare prices $\mathbf{p} = \{p_1^M, p_2^M, p_1^P, p_2^P\}$ to maximize

$$\Pi(\mathbf{p};\mathbf{r}) = \mu \underbrace{\left(\pi_M(p_1^M; r_1^M) + \delta\pi_M(p_2^M; r_2^M(p_1^M))\right)}_{\text{Medicare market profits}} + (1-\mu)\underbrace{\left(\pi_P(p_1^P;\rho) + \delta\pi_P(p_2^P;\rho)\right)}_{\text{private market profits}} + \delta^2 \underbrace{EV(p_2^M)}_{\text{cont. value}}$$

where $\pi_i(p_t; r_t) \equiv Q_i(\lambda r_t - p_t)(p_t - c)$ are flow profits in period t, and the term $EV(p_2^M)$ captures total discounted continuation profits across Medicare and private market; the continuation value is no longer scaled by the fraction of Medicare patients, μ. To the extent that firms can affect future reimbursement via their second period Medicare price, continuation profits are a function of p_2^M; in theory p_2^P could matter for continuation value also, but that is not relevant for the analysis of Medicare price setting.

Optimal prices will depend on the elasticity of demand with respect to the effective margin under each payer, since the payment a physician receives is the difference between list price and reimbursement. Define the Medicare-specific semi-elasticity of demand with respect to the effective Medicare margin as $\eta_M(m) \equiv \frac{Q_M'(m)}{Q_M(m)}$. The first order conditions for the optimal Medicare prices are then:

$$p_1^M = c + \frac{1}{\eta_M(m_{M1})} + \delta(p_2^M - c)\underbrace{\frac{Q_M'(m_{M2})}{Q_M'(m_{M1})}(1+A)\lambda\mu}_{\substack{\text{effect of} \uparrow p_1 \\ \text{on period 2 demand}}}, \tag{A.6}$$

$$p_2^M = c + \frac{1}{\eta_M(m_{M2})} + \underbrace{\delta\frac{\frac{1}{\mu}EV'(p_2^M)}{Q_M'(m_{M2})}}_{\substack{\text{effect of} \uparrow p_2^M \\ \text{on continuation profits}}}. \tag{A.7}$$

As in the baseline model with a single price, the Medicare pricing decisions are not independent across time because m_{M2} depends on p_1^M; the optimal launch price depends on the period 2 price and vice versa.

Similar to the main text's model, we require four technical assumptions in this version of model to derive our results. First, we assume that the physician puts a positive weight on reimbursement and that the patient out-of-pocket share is constant. Second, we assume that conditions hold such that the pharmaceutical's pricing problem is globally convex. Third, we assume that the impact of period 2 Medicare price on continuation value profits is not too negative. Fourth, we assume that the firm cannot make infinite profits in the future by raising current prices (e.g. the continuation value of future profits is not convex in the period two Medicare price).

Explicitly, we assume:

(i) effective margin is increasing in reimbursement ($\lambda > 0$), and the patient out-of-pocket share is constant, $\frac{oop(r_t)}{r_t} = k \ \forall r_t$ for some $k \in \mathbb{R}^+$;

(ii) that demand is weakly concave in the effective margin $Q''_M(m_{Mt}) \leq 0$;

(iii) that the effect of p_2 on continuation profits is non-convex, such that $EV''(p_2^M) \leq 0$;

(iv) and that the second order conditions hold so that the first-order conditions characterize the optimal prices.

Proposition A.1 now provides the necessary and sufficient condition for when lagged-price reimbursement will lead to a declining *Medicare* price path. Echoing the intuition from the baseline model, as long as the semi-elasticity of demand in period 2 is not too much more inelastic, price will decline over time. The larger the ASP add-on $(1 + A)$, the discount rate, and the share of Medicare patients, the more semi-elasticity of demand can differ.

Proposition A.1 *The equilibrium Medicare price will decrease over time ($p_1^M > p_2^M$) if and only if:* $\frac{\eta_M(m_{M1})}{\eta_M(m_{M2})} < \frac{1}{1+\delta\frac{\frac{1}{\mu}EV'(p_2^M)}{Q_M(m_{M2})}} + \delta(1 + A)\lambda\mu\frac{Q_M(m_{M2})}{Q_M(m_{M1})}$.

Proof. The difference between the optimal Medicare prices in period 2 and period 1 from equations (1) and (2) can be written as:

$$p_2^M - p_1^M = \frac{1}{\eta_M(m_{M2})}\left[1 - \delta\frac{Q'_M(m_{M2})}{Q'_M(m_{M1})}(1 + A)\lambda\mu\right] - \frac{1}{\eta_M(m_{M1})} + \delta\frac{\frac{1}{\mu}EV'(p_2^M)}{Q'_M(m_{M2})}\left[1 - \delta\frac{Q'_M(m_{M2})}{Q'_M(m_{M1})}(1 + A)\lambda\mu\right]$$

This is negative if and only if $\frac{1}{\eta_M(m_{M2})}\left(1-\delta\frac{Q'_M(m_{M2})}{Q'_M(m_{M1})}(1+A)\lambda\mu\right)\left(1+\delta\frac{\frac{1}{\mu}EV'(p_2^M)}{Q_M(m_{M2})}\right) < \frac{1}{\eta_M(m_{M1})}$, where we factored out $\frac{1}{\eta_M(m_{M2})}$. Given that $\eta_M > 0$, and that $\frac{Q'_M(m_{M2})}{Q'_M(m_{M1})} = \frac{\eta_M(m_{M2})Q_M(m_{M2})}{\eta_M(m_{M1})Q_M(m_{M1})}$, we can rearrange to yield the condition $\left(1+\delta\frac{\frac{1}{\mu}EV'(p_2^M)}{Q_M(m_{M2})}\right) < \frac{\eta_M(m_{M2})}{\eta_M(m_{M1})}\left(1+\delta(1+A)\lambda\mu\frac{Q_M(m_{M2})}{Q_M(m_{M1})}\left(1+\delta\frac{\frac{1}{\mu}EV'(p_2^M)}{Q_M(m_{M2})}\right)\right)$.

We can see that $\left(1+\delta\frac{\frac{1}{\mu}EV'(p_2^M)}{Q_M(m_{M2})}\right) \geq 0$ by construction. As a result, we can further rearrange to yield the condition
$\frac{\eta_M(m_{M1})}{\eta_M(m_{M2})} < \left(1+\delta(1+A)\lambda\mu\frac{Q_M(m_{M2})}{Q_M(m_{M1})}\left(1+\delta\frac{\frac{1}{\mu}EV'(p_2^M)}{Q_M(m_{M2})}\right)\right)/\left(1+\delta\frac{\frac{1}{\mu}EV'(p_2^M)}{Q_M(m_{M2})}\right)$. ■

B.2.1 Quantity Weighted Price

When the Medicare price is declining, it is clear that the average price (weighted by the share of the population on Medicare) will decline when the private price is constant. However, the quantity weighted price is more complicated: it is also the case that the Medicare market share is changing over time. Moreover, even though the Medicare price is falling, because quantity depends on price relative to reimbursement, it may not be the case that Medicare market share rises over time.

Here, we examine the quantity weighted price. Let the equilibrium Medicare market share in period t be $s_t = \frac{\mu Q_{Mt}}{\mu Q_{Mt}+(1-\mu)Q_{Pt}}$, and then let the quantity weighted average sales price be $\tilde{p_t} \equiv s_t p_t^M + (1-s_t)p_t^P$, where p_t^P denotes the private market price. When both the private reimbursement is constant over time ($p_t^P = p^P$) and the Medicare market share is constant over time ($s_t = s$), then quantity weighted price clearly declines over time ($\tilde{p_2} - \tilde{p_1} < 0$) when the Medicare price declines. Proposition A.2 shows how much s_t can vary over time and still have quantity weighted price decline. We think the most empirically relevant case is case (a), which assumes that the private price is higher than the Medicare price, since private insurance tends to set higher reimbursement rates than Medicare (Government Accountability Office 2016), thus allowing pharmaceutical firms to charge higher prices to physicians who treat privately insured patients.

Proposition A.2 *Assume the private price is constant over time: $p_t^P = p^P$. The quantity weighted average sales price declines over time ($\tilde{p_2} - \tilde{p_1} < 0$) if:*

(a) the private price exceeds the Medicare price in the first period ($p^P > p_1^M$) and Medicare market share is weakly increasing such that shares satisfy $\frac{s_1}{s_2} \leq \left(1-\frac{p_2^M}{p^P}\right)/\left(1-\frac{p_1^M}{p^P}\right)$;

(b) the Medicare price exceeds the private price in the first period ($p_1^M > p^P$) and Medicare market share is weakly decreasing such that shares satisfy $\frac{s_1}{s_2} \geq \left(1-\frac{p_2^M}{p^P}\right)/\left(1-\frac{p_1^M}{p^P}\right)$;

(c) the Medicare price in the first period exceeds the private price, and both exceed the Medicare price in the second period ($p_1^M > p^P > p_2^M$).

Proof. To begin, first note that the difference in the quantity weighted average sales price can be written as: $\tilde{p_2} - \tilde{p_1} = s_2 p_2^M - s_1 p_1^M + (s_1 - s_2)p^P$. Divide by p^P and rearrange. Then, the quantity weighted average sales price is declining iff:

$$\tilde{p_2} - \tilde{p_1} \propto s_1 \left(1 - \frac{p_1^M}{p^P}\right) - s_2 \left(1 - \frac{p_2^M}{p^P}\right) < 0 \tag{A.8}$$

In case (a), $p^P > p_1^M$ implies that $\left(1 - \frac{p_1^M}{p^P}\right) > 0$. It then follows that $p^P > p_1^M$ and condition $\frac{s_1}{s_2} < \left(1 - \frac{p_2^M}{p^P}\right) / \left(1 - \frac{p_1^M}{p^P}\right)$ jointly imply that the key equation above holds.

In case (b), $p_1^M > p^P$ implies that $\left(1 - \frac{p_1^M}{p^P}\right) < 0$. It follows that $p_1^M > p^P$ and condition $\frac{s_1}{s_2} > \left(1 - \frac{p_2^M}{p^P}\right) / \left(1 - \frac{p_1^M}{p^P}\right)$ jointly imply that the key equation above holds

Finally, in case (c), $p_1^M > p^P$ implies that $\left(1 - \frac{p_1^M}{p^P}\right) < 0$. Since $p^P > p_2^M$ implies $\left(1 - \frac{p_2^M}{p^P}\right) > 0$, the key equation above holds. ■

C Empirical Appendix

Table A1: Effect of MMS on Price Growth: Early Versus Late Cohorts

	(1)	(2)	(3)	(4)
	Early Cohort (2005-2011)		Late Cohort (2012-2019)	
Quarters Since Launch (τ)	0.004		0.008***	
	(0.007)		(0.002)	
MMS × Quarters Since Launch (τ)	-0.007		-0.011***	
	(0.008)		(0.004)	
Quarters Since Launch (τ) > 12		0.112		0.057**
		(0.071)		(0.027)
Above Median MMS × Quarters Since Launch (τ) > 12		-0.152*		-0.062**
		(0.087)		(0.031)
Drug Fixed Effects	Yes	Yes	Yes	Yes
Year Fixed Effects	Yes	Yes	Yes	Yes
R^2	0.072	0.104	0.199	0.171
N	2945	2945	1557	1557

Notes: *** $p < 0.01$, ** $p < 0.05$, * $p < 0.1$. Dependent variable: $\ln p_{jt}$. Data: Analysis Sample, split by cohort of introduction, as indicated. Robust standard errors clustered at the HCPCS level.

Table A2: Effect of MMS on Price Growth: Drug Categories

	(1)	(2)	(3)	(4)	(5)	(6)
	Chemotherapy		Injections		Other	
Quarters Since Launch (τ)	-0.012		0.010***		0.011	
	(0.014)		(0.003)		(0.021)	
MMS × Quarters Since Launch (τ)	0.038		-0.013***		-0.009	
	(0.026)		(0.004)		(0.019)	
Quarters Since Launch (τ) > 12		0.029		0.053*		0.010
		(0.023)		(0.030)		(0.075)
Above Median MMS × Quarters Since Launch (τ) > 12		-0.005		-0.080*		-0.021
		(0.031)		(0.042)		(0.034)
Drug Fixed Effects	Yes	Yes	Yes	Yes	Yes	Yes
Year Fixed Effects	Yes	Yes	Yes	Yes	Yes	Yes
R-squared	0.421	0.393	0.242	0.211	0.170	0.161
N	985	985	2816	2816	701	701

Notes: *** $p < 0.01$, ** $p < 0.05$, * $p < 0.1$. Dependent variable: $\ln p_{jt}$. Data: Analysis Sample. Robust standard errors clustered at the HCPCS level.

Table A3: Descriptive Statistics, Split By Private Market Share of Older Individuals

	Full Sample		Above Median PMSOI	Below Median PMSOI
	Mean	*Std Dev*	*Mean*	*Mean*
Private Market Share of Older Individuals (PMSOI) at $\tau = 1$	0.591	0.326	0.866	0.319
Relative ASP at $\tau = 8$	1.041	0.293	1.022	1.059
Average Year of Introduction	2010.9	3.8	2011.3	2010.5
Compound Annual Growth Rate over first 6 years	0.0382	0.0933	0.0247	0.0505
Compound Quarterly Growth Rate over first 6 years	0.0086	0.0227	0.0053	0.0117
N (Unique HCPCS)	197		98	99

Notes: Source: Authors' calculations from CMS Data 2006-2019 and aggregate Truven Marketscan spending by age. "Private Market Share of Older Individuals" (PMSOI) is created for each HCPCS-year as the total revenue for the older age category (age 56 to 64) over the total revenue for the older and younger (26 to 44 years old) age categories summed. Number of observations is lower than in the Analysis Sample due to missing data (HCPCS with no private spending). Median Private Market Share of Older Individuals at Launch = .69

Table A4: Private Market Share of Older Individuals (PMSOI) and Price Growth

	(1)	(2)
Quarters Since Launch (τ)	0.020***	
	(0.005)	
PMSOI × Quarters Since Launch (τ)	-0.021***	
	(0.006)	
Quarters Since Launch (τ) > 12		0.081**
		(0.033)
Above Median PMSOI × Quarters Since Launch (τ) > 12		-0.134**
		(0.057)
Drug Fixed Effects	Yes	Yes
Year Fixed Effects	Yes	Yes
R^2	0.111	0.105
N	4080	4080

Notes: *** $p < 0.01$, ** $p < 0.05$, * $p < 0.1$. Dependent variable: $\ln p_{jt}$. Data: Analysis Sample. Robust standard errors clustered at the HCPCS level.

Figure A2: Medicare Quantity Sold, Relative to Launch Period

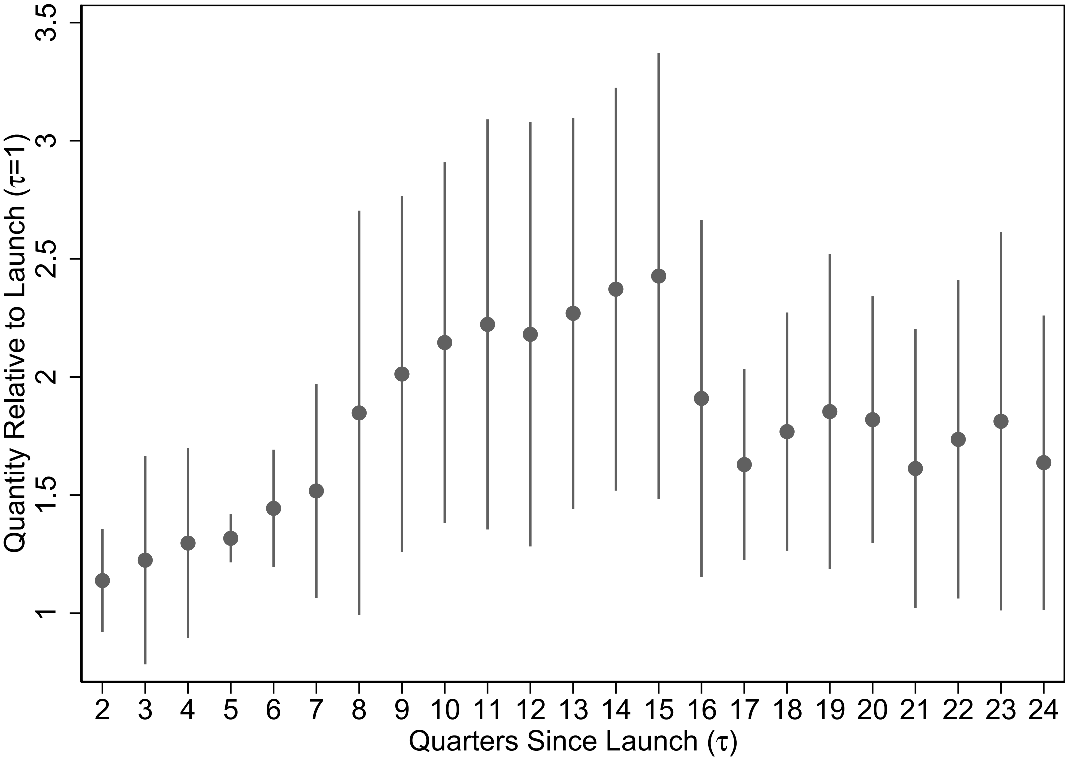

Notes: Data: Analysis Sample. Quantity in each quarter is calculated as total Medicare revenue divided by ASP. Relative quantity is quantity in quarter τ divided by quantity in quarter $\tau = 1$. Relative quantity is winsorized at the 1st and 99th percentiles due to outliers. Plots the results of a regression of relative quantity against quarter τ fixed effects and year-quarter fixed effects weighted by total drug market size. Point estimates and 95% confidence intervals of the quarter τ fixed effects are present. Standard errors clustered at the HCPCS level. Note that 2 years after launch, median quantity sold is very similar to quantity at launch (relative quantity=0.99), while the 99th percentile of relative quantity is over 20. This accounts for the jump in standard errors in the figure beginning 2 years after launch.

Figure A3: Distribution of MMS at Launch

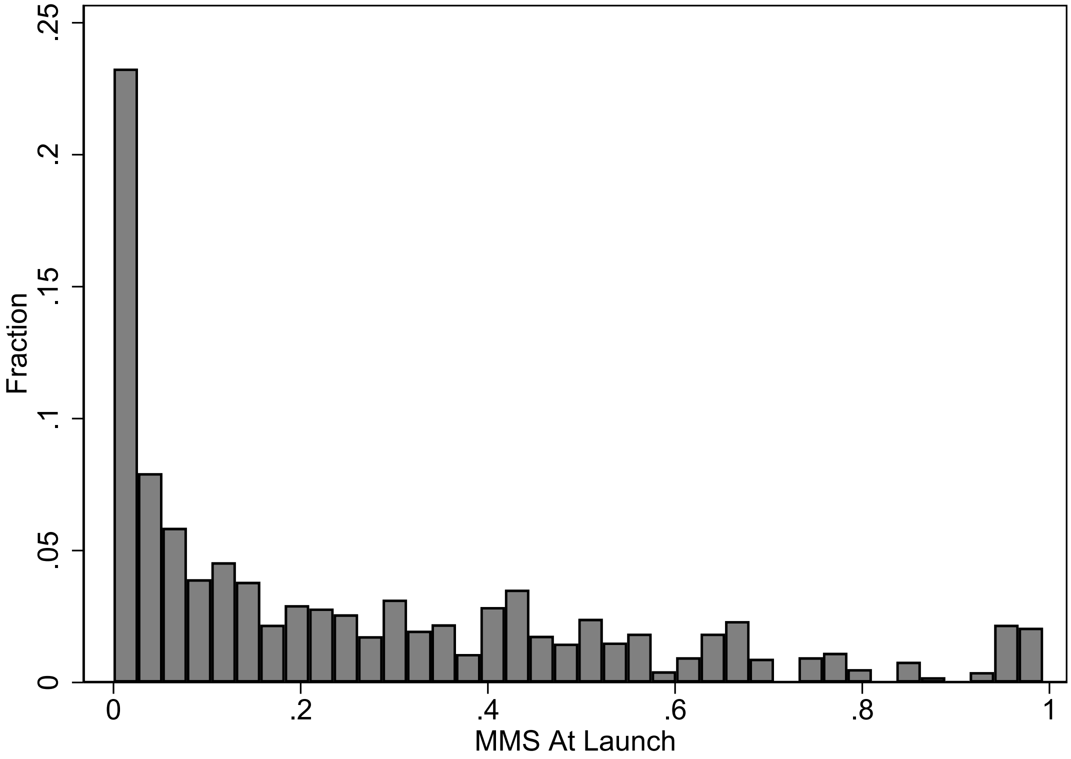

Notes: Data: Analysis Sample

Figure A4: Time Until Competitor Entry in Billing Code

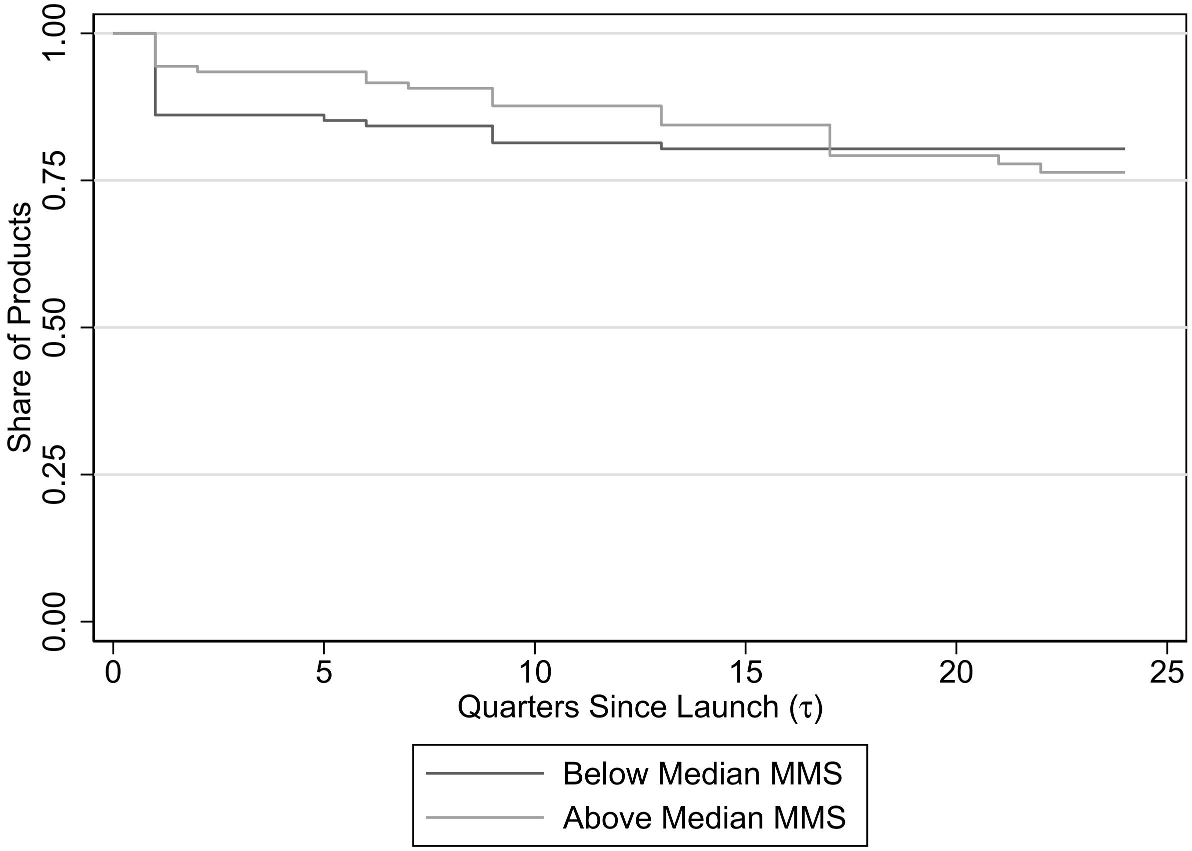

Notes: Plots Kaplan-Meier survivor function for being billing code monopolist split by above versus below median Medicare market share. Early entry due in part to new J-Codes that have old products. In Cox proportional hazard model, above median MMS products are more likely to have entry, but this difference is not statistically significant (Hazard ratio 0.975, 95% CI 0.54 to 1.75). Data: Analysis Sample.

Figure A5: Time Until Competitor Entry in Billing Code: Sample of Newly Approved Molecules

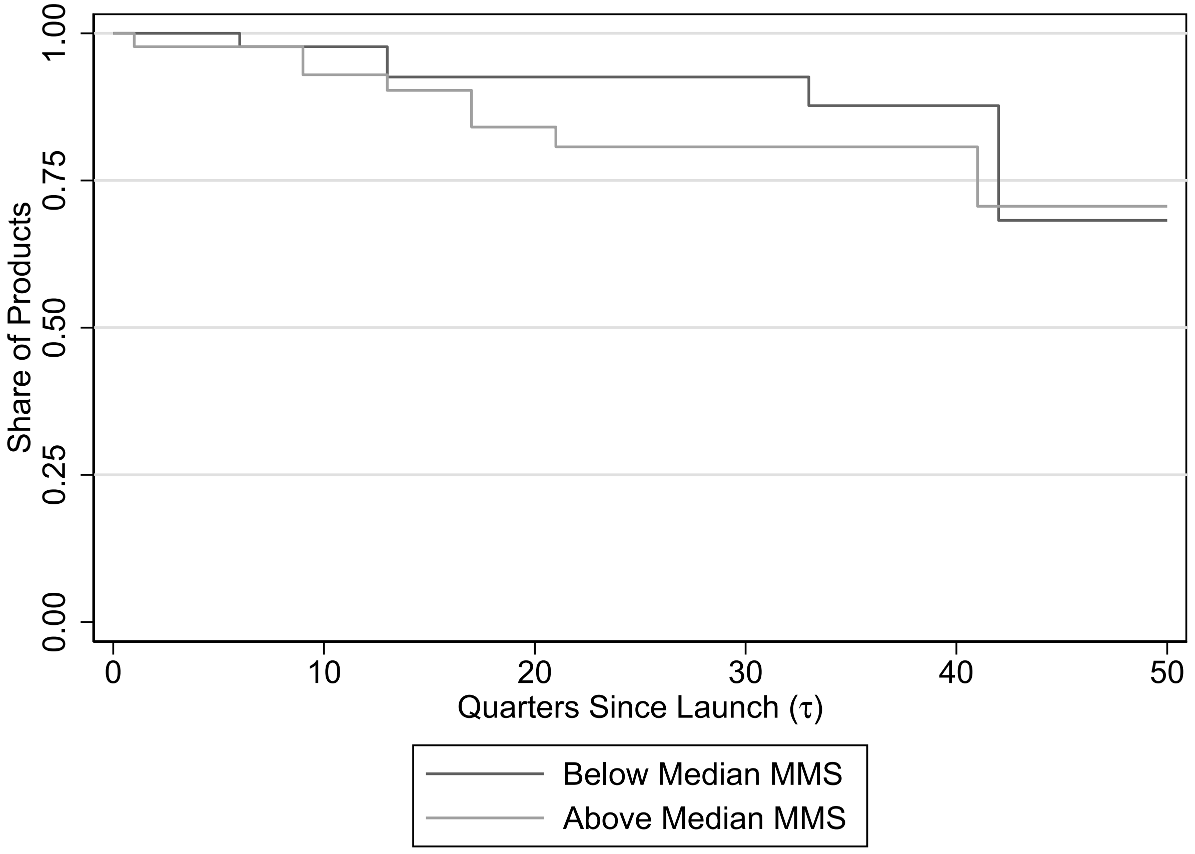

Notes: Plots Kaplan-Meier survivor function for being billing code monopolist.

Figure A6: Robustness Checks

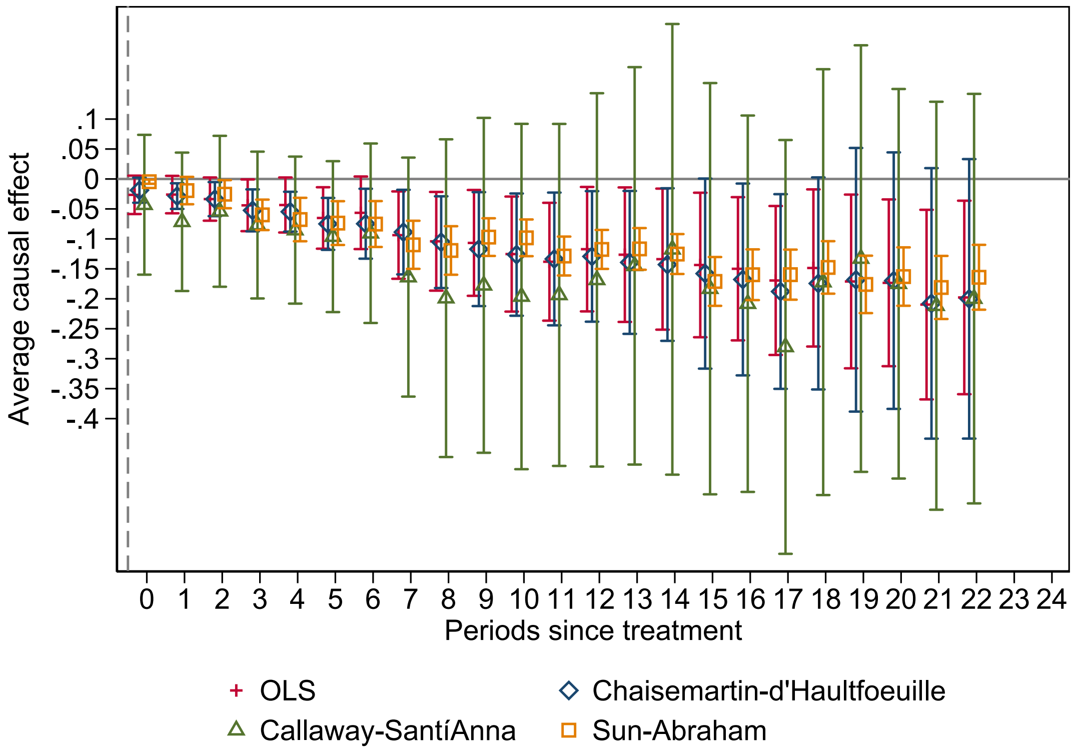

Notes: Plots point estimates and 95% confidence intervals for coefficients from four different estimators. OLS is estimated by regression Equation 3 in which treatment is discretized into above versus below median MMS. Then, results from three additional two-way fixed effects estimators are presented: Callaway and Sant'Anna (2021), Chaisemartin and d'Haultfoeuille (2020), and Sun and Abraham (2021). Data: Analysis Sample. Robust standard errors clustered at the HCPCS level.

Figure A7: Robustness Checks

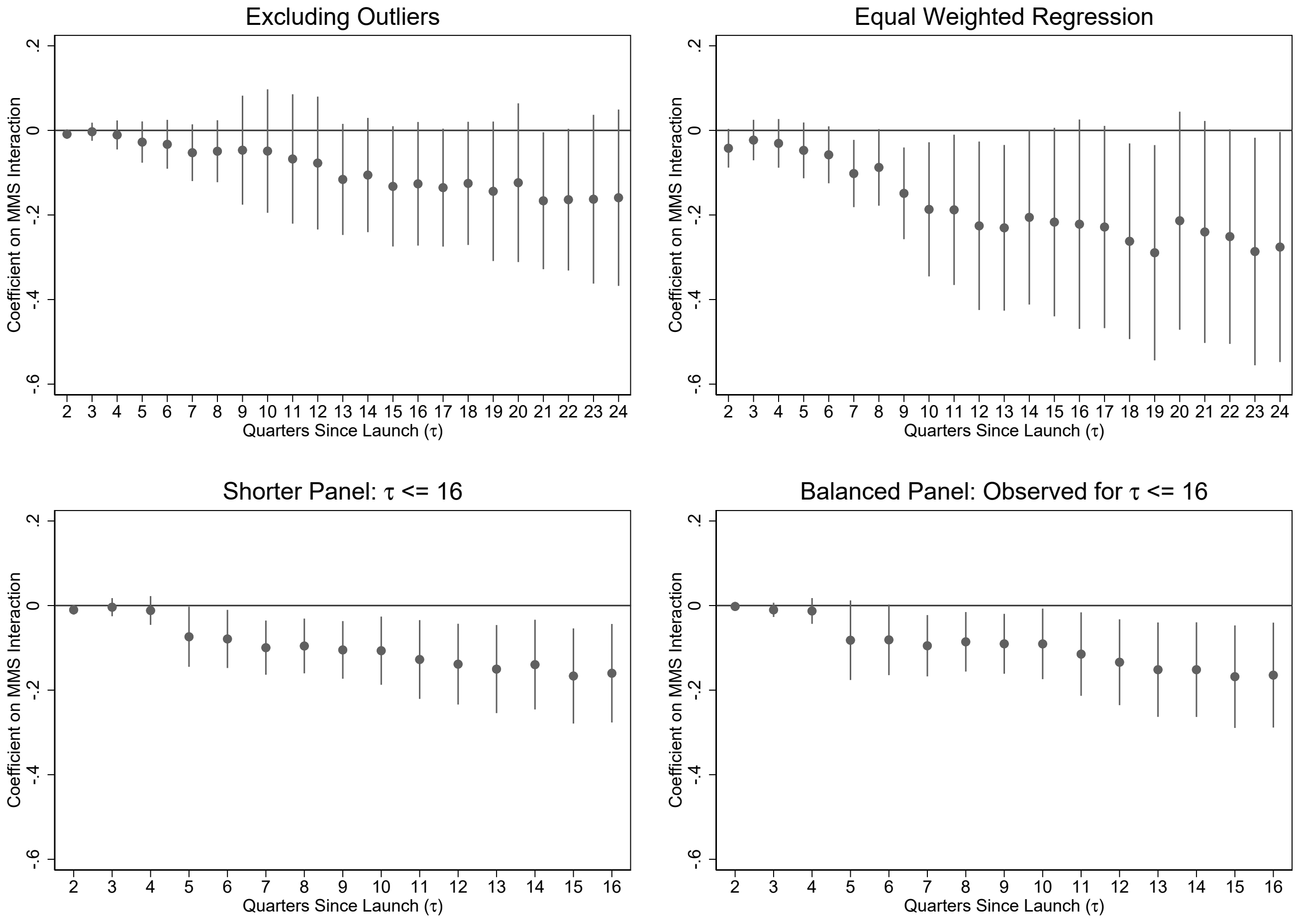

Notes: Plots point estimates and 95% confidence intervals for coefficients estimated by regression Equation 3. Data: Analysis Sample with modifications as shown in each subfigure's title. Robust standard errors clustered at the HCPCS level.

Figure A8: Additional Robustness Checks

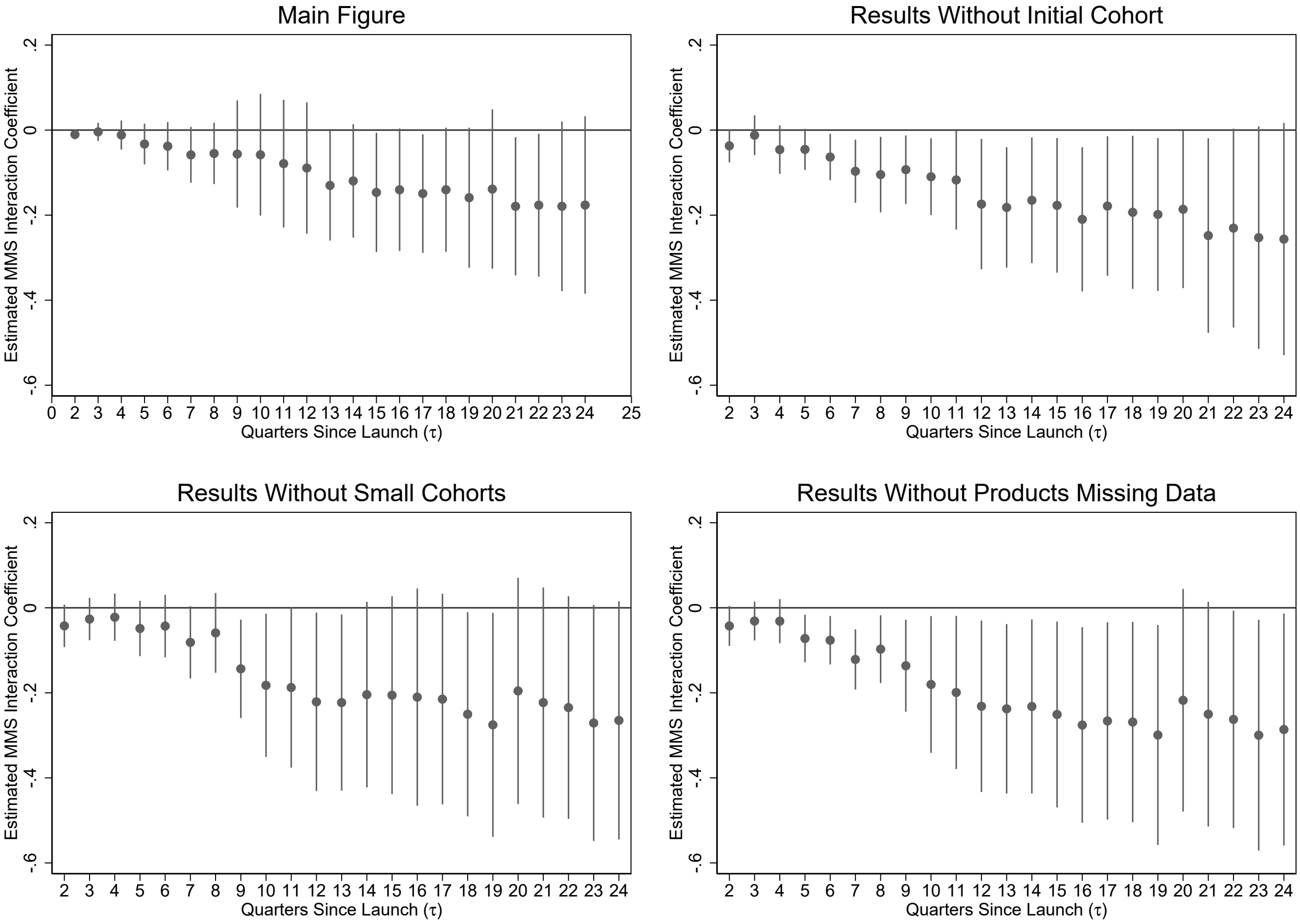

Notes: Plots point estimates and 95% confidence intervals for coefficients estimated by regression Equation 3. Data: Analysis Sample, with modifications as follows. Main Figure recreates the estimates of Figure 3 on a common axis for reference. Results "Without Initial Cohort" estimates the effects without the earliest (and largest) treatment cohort (7.9% of observations). Results "Without Small Cohorts" estimates the effects without cohorts of two or fewer products (3.8% of observations). Results "Without Products Missing Data" estimates the effects without products which are missing any quarter of data (6.8% of observations). Robust standard errors clustered at the HCPCS level.
